# Proviral location affects cognate peptide–induced virus production and immune recognition of HIV-1–infected T cell clones

**DOI:** 10.1172/JCI171097

**Published:** 2023-11-01

**Authors:** Filippo Dragoni, Abena K. Kwaa, Caroline C. Traut, Rebecca T. Veenhuis, Bezawit A. Woldemeskel, Angelica Camilo-Contreras, Hayley E. Raymond, Arbor G. Dykema, Eileen P. Scully, Amanda M. Rosecrans, Kellie N. Smith, Frederic D. Bushman, Francesco R. Simonetti, Joel N. Blankson

**Affiliations:** 1 Department of Medicine,; 2 Department of Molecular and Comparative Pathobiology, and; 3 Department of Neurology, Johns Hopkins University School of Medicine, Baltimore, Maryland, USA.; 4 Department of Microbiology, University of Pennsylvania, Perelman School of Medicine, Philadelphia, Pennsylvania, USA.; 5 Bloomberg~Kimmel Institute for Cancer Immunotherapy, and; 6 Sidney Kimmel Comprehensive Cancer Center, Johns Hopkins University School of Medicine, Baltimore, Maryland, USA.

**Keywords:** AIDS/HIV, Cellular immune response

## Abstract

**BACKGROUND:**

HIV-1–infected CD4^+^ T cells contribute to latent reservoir persistence by proliferating while avoiding immune recognition. Integration features of intact proviruses in elite controllers (ECs) and people on long-term therapy suggest that proviruses in specific chromosomal locations can evade immune surveillance. However, direct evidence of this mechanism is missing.

**METHODS:**

In this case report, we characterized integration sites and full genome sequences of expanded T cell clones in an EC before and after chemoradiation. We identified the cognate peptide of infected clones to investigate cell proliferation and virus production induced by T cell activation, and susceptibility to autologous CD8^+^ T cells.

**RESULTS:**

The proviral landscape was dominated by 2 large clones with replication-competent proviruses integrated into zinc finger (ZNF) genes (*ZNF470* and *ZNF721*) in locations previously associated with deeper latency. A third nearly intact provirus, with a stop codon in Pol, was integrated into an intergenic site. Upon stimulation with cognate Gag peptides, infected clones proliferated extensively and produced virus, but the provirus in *ZNF721* was 200-fold less inducible. While autologous CD8^+^ T cells decreased the proliferation of cells carrying the intergenic provirus, they had no effect on cells with the provirus in the *ZNF721* gene.

**CONCLUSIONS:**

We provide direct evidence that upon activation of infected clones by cognate antigen, the lower inducibility of intact proviruses in ZNF genes can result in immune evasion and persistence.

**FUNDING:**

Office of the NIH Director and National Institute of Dental & Craniofacial Research; NIAID, NIH; Johns Hopkins University Center for AIDS Research.

## Graphical abstract

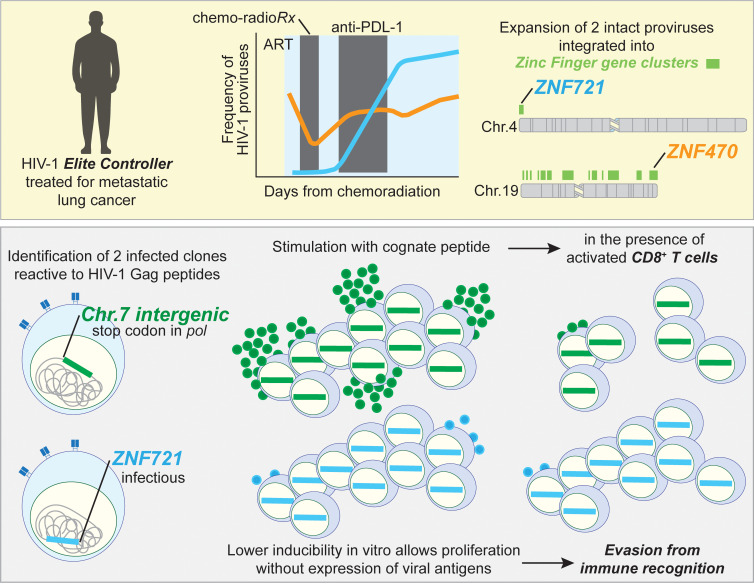

## Introduction

Shortly after transmission, HIV-1 infection establishes a persistent reservoir of latently infected CD4^+^ T cells (1–4). Although antiretroviral therapy halts viral replication and prevents disease progression, the long half-life of HIV-1 reservoir cells (*t*_1/2_ greater than approximately 44 months) results in viral rebound upon treatment interruption in most individuals (5, 6). Elite controllers (ECs) can spontaneously restrict viral replication in the absence of antiretroviral therapy (ART) due to highly functional cytolytic immune responses that rapidly clear productively infected cells, resulting in undetectable viral loads (7–9). These individuals (<1% of people living with HIV-1 [PLWH]), represent the ultimate goal of therapeutic strategies aimed at HIV-1 remission (10, 11).

In ECs and people on ART, reservoir stability is mostly caused by the proliferation of infected cells, which undergo clonal expansion beginning in the earliest stages of HIV-1 infection, driven by encounter with cognate antigens and survival stimuli (IL-2, IL-7, and IL-15) (12–21). In addition, HIV-1 integration into a handful of specific genes causes insertional mutagenesis, providing a survival advantage (22, 23). Thus, therapeutic strategies that disrupt the proliferation of infected cells would significantly accelerate reservoir decay, contributing to the success of future curative interventions (24). However, cytoreductive and antiproliferative compounds tested so far failed to significantly reduce reservoir size; moreover, current knowledge of the impact of antiproliferative drugs on the heterogeneous population of HIV-1–infected clones persisting on ART is limited (25, 26).

The proviral landscape is dynamic, shaped by selective forces imposed by HIV-1–host interactions. Growing evidence suggests that HIV-1 integration into chromosomal locations associated with deeper latency are positively selected over time in both ECs and PLWH on ART (18, 27, 28). Such genomic regions include centromeric and pericentromeric repeats, lamina-associated domains (LADs), and the zinc finger (ZNF) gene family. Recent studies suggest that intact proviruses integrated into certain ZNF genes, often recurring across individuals, have a survival advantage due to reduced viral expression caused by the repressive epigenetic environment typical of ZNF gene clusters found in heterochromatic regions (29, 30). However, direct evidence for this mechanism, and whether it affects latency reversal upon physiological stimulation, is lacking. ZNF genes and other repressive genomic locations have been linked to a survival advantage and deeper latency of intact proviruses. In a study from Halvas and colleagues, 2 proviruses located in *ZNF721/ABCA11P* could be recovered from viral outgrowth and 1 contributed to persistent nonsuppressible viremia (31). This signature for reservoir selection was first identified in ECs, likely due to the strong cytotoxic T lymphocyte (CTL) pressure that selects against proviruses with higher transcriptional activity (18, 27). Similarly, more recent studies in people on ART found a progressive enrichment over time of intact proviruses located in these epigenetically repressed regions (28, 29). Whether these proviruses are permanently “blocked and locked,” and the clinical consequences, remain unclear.

In this study, we characterized HIV-1 persistence in an HLA-B*57^+^ EC before and after treatment with ART, chemoradiation, and anti–PD-L1 immunotherapy for metastatic lung cancer. We performed longitudinal integration site analysis and quantified intact and total HIV-1 DNA by digital PCR (dPCR). We also assessed the clonal dynamics of 2 intact proviruses integrated into ZNF genes that, by the end of treatment, dominated the reservoir. Finally, by PBMC stimulation experiments, we resolved the T cell specificity at the epitope level of 2 infected clones, which allowed us to dissect cell proliferation and provirus inducibility upon peptide–MHC class II recognition, and the impact of immune recognition by autologous CD8^+^ T cells.

## Results

### Chemoradiation induces a transient contraction of HIV-1–infected cells.

**Figure 1:**
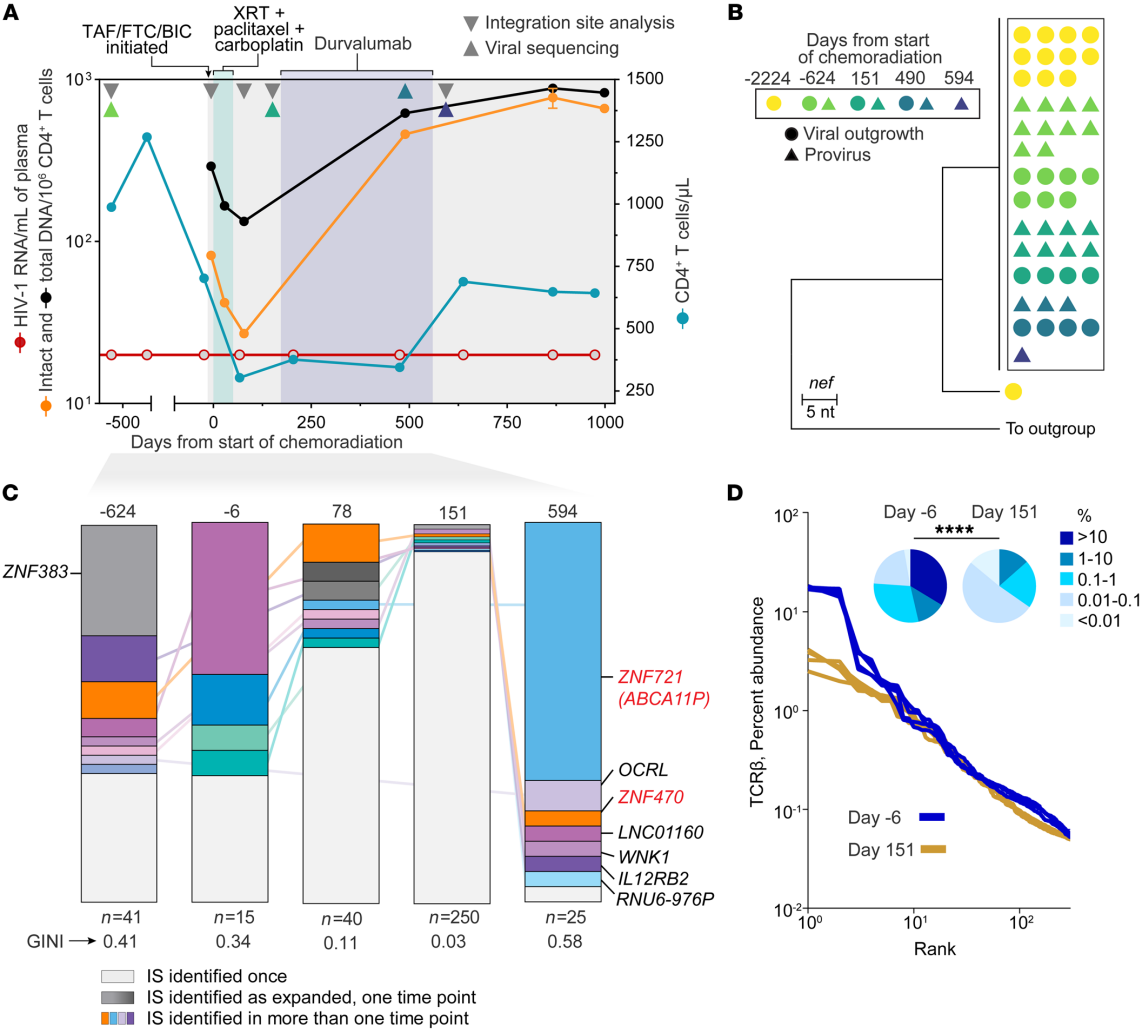
Chemoradiation and immunotherapy transiently affect the HIV-1 reservoir cell frequency and composition. (**A**) Clinical history of study participant ES24 before, during, and after treatment of metastatic lung cancer; values below the limit of detection are indicated with open symbols; intact and total proviruses in total CD4^+^ T cells were quantified by IPDA (orange and black, respectively). TAF, tenofovir alafenamide; FTC, emtricitabine; BIC, bictegravir; CRT, chemoradiation therapy. (**B**) Maximum likelihood tree of *nef* sequences recovered over time from viral outgrowth assays or limiting dilution PCR from CD4^+^ T cell–derived genomic DNA; identical sequences are indicated by the black box; HXB2 was used as outgroup. (**C**) Longitudinal integration site analysis shows contraction and reconstitution of proviruses in expanded clones; total integration sites recovered in each sample are indicated below stacked bars; integrations in ZNF genes relevant in subsequent analyses are highlighted in red. IS, integration site. (**D**) Chemoradiation perturbs the CD4^+^ T cell repertoire, especially causing loss of most expanded clones; rank-abundance curves indicate distribution of clonality among the top 250 TCRβ sequences; pie charts indicate distribution of clonotypes based of relative abundance. Significance of differences between the 2 time points was assessed by χ^2^ test. ****P < 0.0001.

The study participant, here referred to as ES24, is a previously described HLA-B*57^+^ male EC who maintained undetectable HIV-1 RNA without treatment for more than 10 years (15). In 2019, he was diagnosed with metastatic lung cancer for which he initiated ART and was treated with surgery and a cycle of 5 weekly doses of carboplatin and paclitaxel chemotherapy with radiotherapy (chemoradiation therapy or CRT). This was followed by a 12-month course of immunotherapy with 12 cycles of anti–PD-L1 monoclonal antibody treatment (durvalumab, additional details on his clinical history are provided in Figure 1A). Of note, CD4^+^ T cells reached a nadir on day 67 after CRT (304 cells/μL, 28.8%) and partially recovered by the end of the study (643 cells/μL, 30.4%, on day 974). To investigate the impact of treatment on reservoir dynamics, we measured total and intact HIV-1 DNA longitudinally before, during, and after CRT (Figure 1A). We observed a rapid but transient decline in both total and intact DNA (2.2- and 3.0-fold reduction, respectively) at 1 month after CRT that coincided with the CD4^+^ T cell count nadir (2.3-fold reduction). However, HIV-1 DNA increased in the months after treatment, with a rebound from nadir that was markedly higher for intact DNA (3- versus 9.4-fold for total and intact DNA, respectively), which reached higher levels than pre-CRT (663 versus 82 copies/10^6^ CD4^+^ T cells). Interestingly, single genome sequencing of proviral DNA and outgrowth virus showed a single dominant variant that did not evolve during the study period (Figure 1B), suggesting that the persistence of reservoir cells was driven by cell survival and proliferation, rather than low-level replication, also reflected by the persistently undetectable viral load in plasma. Thus, to further characterize changes in infected cell composition we performed integration site analysis (Figure 1C), which revealed a transient contraction in clonality during the CD4 nadir (Gini index 0.41, 0.03, 0.58 on day –624, 151, and 594, respectively). Of note, some integration sites were detected at multiple time points, including a provirus found in the *ZNF721* gene, which underwent significant expansion from day 78 to day 594 (*P* < 0.0001). Finally, we observed a similar clonal contraction in all CD4^+^ T cells, measured by T cell receptor (TCR) sequencing, which preferentially affected the most abundant clones (Figure 1D), as previously described (32). Overall, these result show that although CRT perturbed the frequency and composition of infected cells, reservoir size rebounded upon CD4^+^ T cell recovery, as recently reported in the context of immunosuppressive therapy after kidney transplant (33).

### Clonal replacement of 2 intact proviruses integrated into ZNF genes.

**Figure 2:**
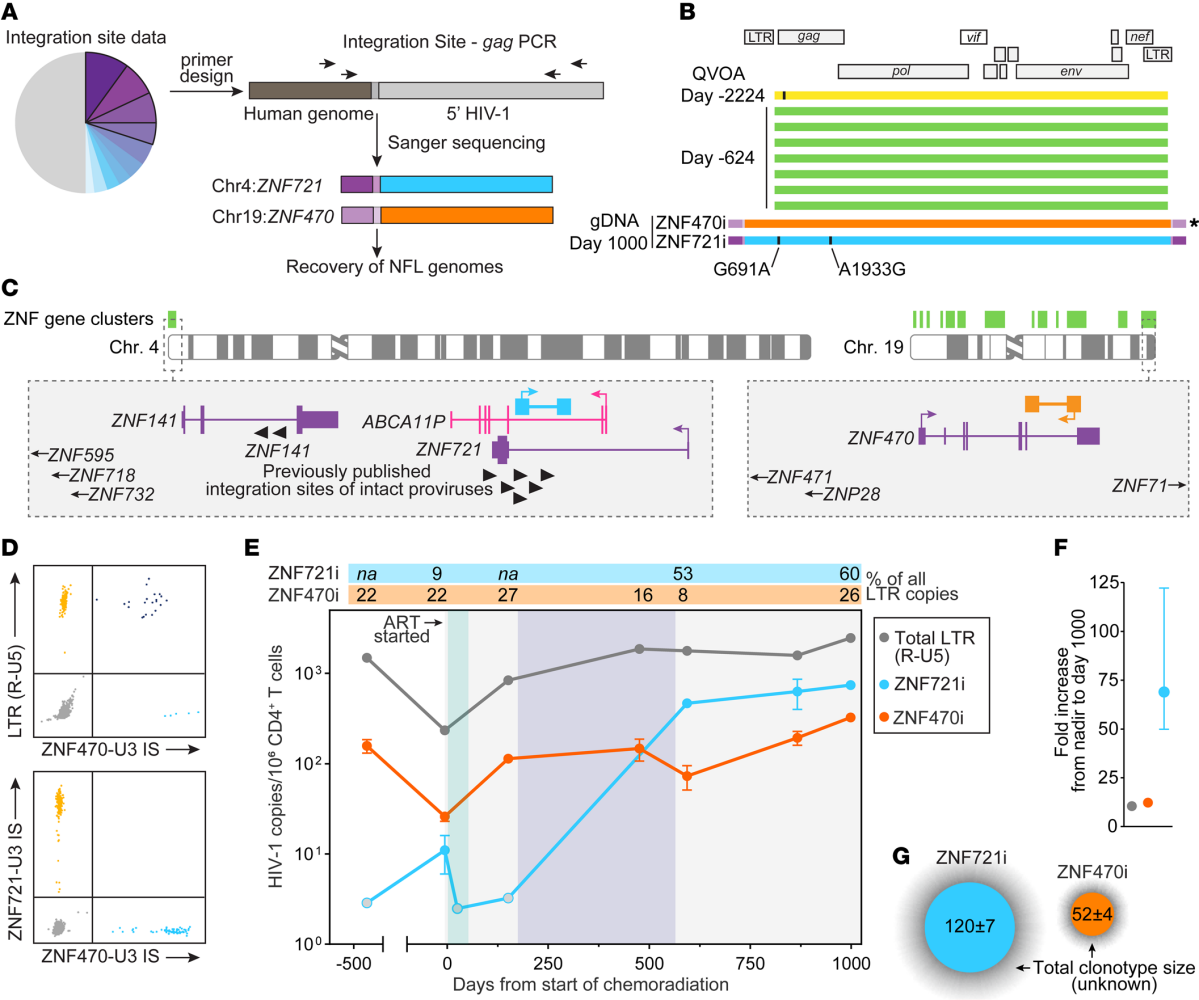
Persistence and expansion of 2 clones carrying intact proviruses integrated in ZNF genes. (**A**) Experimental approach used to match integration site and proviral sequences of interest. (**B**) Nearly full-length (NFL) genome sequences from viral outgrowth isolates and intact proviruses integrated into *ZNF721* and *ZNF470* genes; black ticks show nucleotide differences from ZNF470i, indicated by the asterisk symbol; mutations in ZNF721i relative to ZNF470i are indicated with their positions relative to HXB2. (**C**) Genomic location and relative orientation of intact proviruses of interest; purple tracks indicate protein-coding genes, with the psudogene ABCA11P indicated in pink; black arrow heads indicate previously published integration sites; genes located outside the gray box are highlighted with arrows. (**D**) Representative 2D dPCR plots showing duplex amplification of total LTR R-U5 copies and proviruses of interest by integration site–specific assays. IS, integration site. (**E**) Longitudinal quantification of ZNF721i and ZNF470i proviruses before, during, and after treatment; the gray, green, and purple shaded areas indicate ART, chemoradiation, and immunotherapy, respectively, as in Figure 1A; open circles indicate values below the limit of detection; error bars indicate SEM; values above the graph area represent the percentage of proviruses of interest among all LTR R-U5 copies. (**F**) Fold increase of total LTR and proviruses of interest from day –6 to day 1000 from the start of chemoradiation. (**G**) Estimates of total-body clone size, expressed as million cells; the shaded gray area represents the uninfected fraction of each clonotype.

The sequencing and integration site data suggested that the increase in reservoir size was due to relatively few clones undergoing proliferation, so we sought to determine the genomic integration sites and full genome sequences of infectious proviruses (Figure 2A). We designed primers in the human genome, paired with primers in HIV-1 *gag*, to screen candidate integration sites from day 529, and identified 2 proviruses integrated into the *ZNF721/ABCA11P* and *ZNF470* genes, termed ZNF721i and ZNF470i going forward, that were genetically intact and matched the *nef* region sequenced from previous time points. ZNF470i was identical to the nearly full-length sequences of viral outgrowth variants recovered from day –624, while ZNF721i differed only by 2 nucleotides (Figure 2B). These 2 ZNF genes are located in chromosomes 4 and 19, respectively, both within ZNF gene clusters where intact proviruses have been previously reported from both ECs and people on long-term ART (Figure 2C and Supplemental Table 1; supplemental material available online with this article; https://doi.org/10.1172/JCI171097DS1) (18, 29, 34–36). To investigate the temporal dynamics of these 2 intact proviruses, we quantified their frequencies by integration site–specific dPCR, and their relative abundance among all infected cells, as recently described (37, 38) (see Methods and Figure 2, D and E). Total long terminal repeat (LTR) copies and ZNF470i decreased upon CRT initiation and returned to baseline levels; in contrast, ZNF721i underwent significant proliferation and reached a frequency of 741 ± 48 copies/10^6^ CD4^+^ T cells, corresponding to a 69-fold increase from its nadir on day –6 (11 ± 5 copies/10^6^ cells) (*P* = 0.0001; Figure 2, E and F). On day 1000 after CRT initiation, ZNF721i and ZNF470i together represented more than 80% of all LTR copies, with estimated total-body clone sizes of 120 and 52 million cells, respectively. Together, our results show that these 2 intact proviruses found in ZNF genes persisted despite profound perturbation of the HIV-1 reservoir and, in the case of ZNF721i, underwent extensive clonal selection.

### Infected clones are common among cells reactive to HIV-1 antigens.

**Figure 3:**
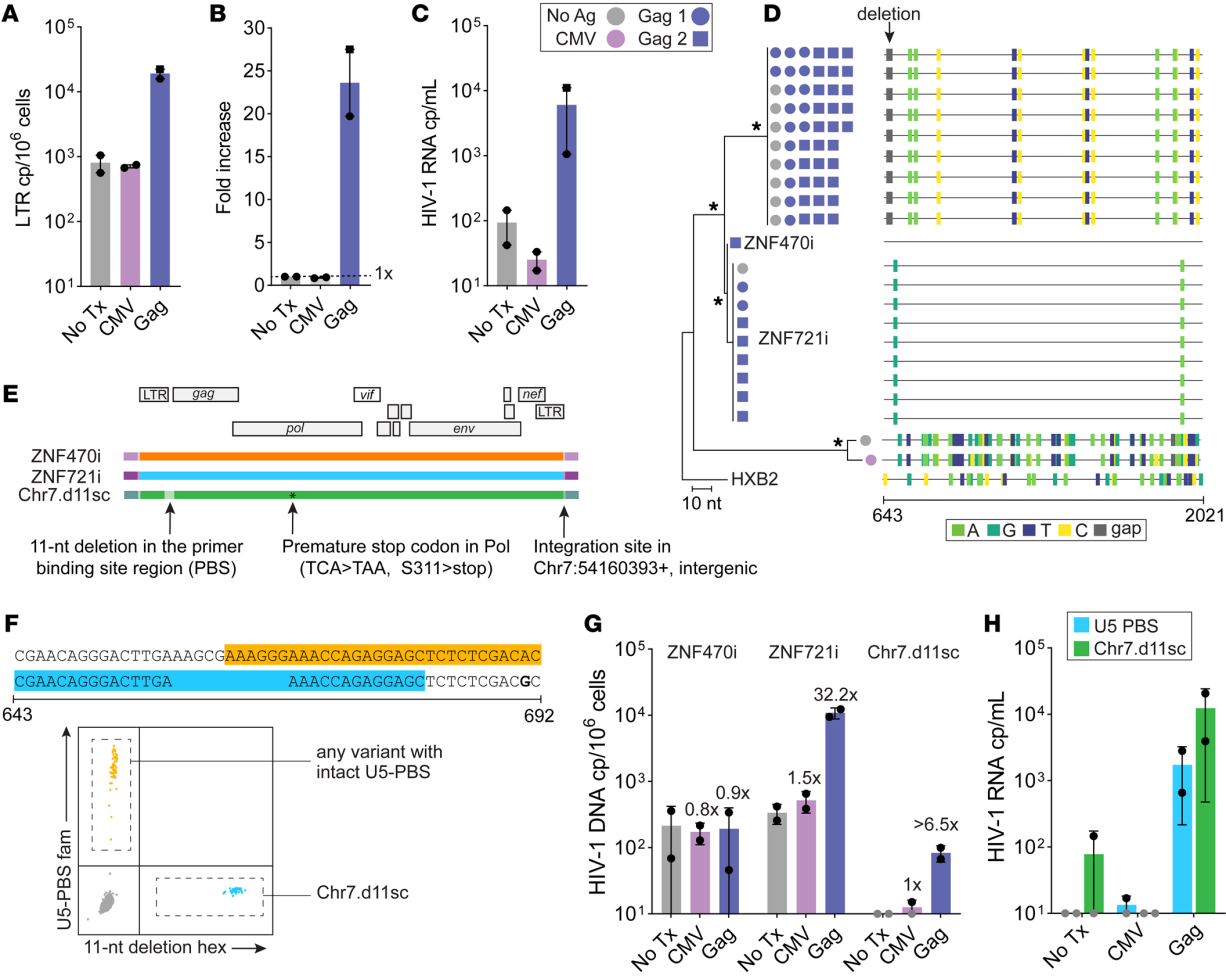
PBMC stimulation with HIV-1 Gag peptides induces proliferation of infected cells and virus production. (**A**) CD8-depleted PBMCs collected on day 926 after CRT were cultured for 9 days in the presence of CMV lysate, a Gag peptide pool, or left untreated (No Tx); total LTR (R-U5) copies were quantified from genomic DNA on day 9. The same Gag peptide pool was used in 2 independent experiments from the same time point. (**B**) Stimulation with Gag peptides led to a marked increase in LTR copies, which was paralleled by virus production detected in the culture supernatant (**C**). (**D**) Maximum likelihood phylogeny analysis of HIV-1 RNA U5-gag single genome sequences; star symbols indicate node with bootstrap >75; black arrow indicates 11-nt deletion; highlighter plot on the right shows nucleotide mutations relative to ZNF470i. (**E**) Characterization of paired full genome sequence and integration site analysis of a new near-intact variant with a premature stop codon in Pol (indicated by asterisk). (**F**) Design of 2 competition probes used to distinguish intact U5-PBS from the Chr7.d11sc variant; the panel at the bottom shows a dPCR 2D plot of a sample containing both targets. (**G**) HIV-1 DNA copies per million cells from day 9 of culture by measuring ZNF470i, ZNF721i, or the 11-nt deletion from Chr7.d11sc; numbers above bar charts indicate fold-change from No Tx. (**H**) HIV-1 RNA copies per mL of supernatant at day 9 of culture; symbols represent 2 independent stimulations; error bars indicate SD.

To investigate the role of antigen-driven selection of infected cells in ES24, we stimulated CD8-depleted PBMCs, in the presence of antiretrovirals, with CMV and HIV-1 antigens (Figure 3), which have been involved in the persistence of HIV-1–infected clones (39–43). Stimulation of PBMCs with a Gag peptide pool led to a significant increase in LTR DNA copies at 9 days (23.6-fold increase, *P* = 0.01; Figure 3, A–C). To determine which proviruses expanded in culture and produced virus, we sequenced the 5′-leader–*gag* region from virion-associated RNA (Figure 3D). We found that 15% of sequences from cells stimulated with Gag peptides matched ZNF721i, while the remaining identified a new variant, markedly different from ZNF721i and ZNF470i, that we did not identify before from proviral DNA or QVOA. This new provirus is nearly intact but shows a premature stop codon in Pol (S311→stop). In addition, it is characterized by an 11-nucleotide deletion within the primer binding site (PBS) hairpin, located in the spacer region between the primer activation sequence and the tRNA annealing region (44) (Figure 3, D and E). The provirus (named Chr7.d11sc because of its features) is located in chromosome 7p12.1, in an intergenic region distinct from those previously associated with deeper latency (30). To investigate the presence of specific histone modifications, we interrogated publicly available ChIP-seq data from CD4^+^ T cells (45); while ZNF721i and ZNF470i were surrounded by epigenetic signatures typical of heterochromatin and ZNF genes (H3K9me3 and H3K36me3), Chr7.d11sc was distant from activating epigenetic marks and showed little enrichment for heterochromatin (Supplemental Figure 1).

To confirm whether the cells carrying this Chr7.d11sc respond to Gag peptide stimulation, we exploited its unique 11-nucleotide deletion to design an assay based on competition probes that would distinguish it from other variants in both genomic DNA and virion-associated RNA (Figure 3F). While ZNF470i did not proliferate in response to antigenic stimulation, we found that exposure to Gag peptides led to virus production and clonal expansion of both ZNF721i and Chr7.d11sc (32- and >6-fold increase, respectively) (Figure 3, G and H). When we compared proviral copies and virus released at the end of culture, we noted that ZNF721i produced less virus despite the extensive proliferation (~10^3^ copies/mL, ~10^4^ copies/10^6^ cells, respectively).

To further characterize these 3 proviruses of interest, we measured their frequency by integration site–specific dPCR and performed a quantitative viral outgrowth assay (qVOA) from CD4^+^ T cells obtained on day 1400 after CRT (Supplemental Figure 2). Out of 5 wells with viral outgrowth (corresponding to 1.44 infectious units per million), 3 had virus matching ZNF470i and 2 wells had virus matching ZNF721i, demonstrating that these 2 proviruses are infectious and inducible despite being located within ZNF genes. However, based on the high number of ZNF470i and ZNF721i proviruses screened in the qVOA (961 and 1490), we estimated that less than 0.3% (95% CI 0.9%–0.08%) of proviruses were induced and resulted in outgrowth in vitro (Supplemental Figure 2). In addition, we quantified cell-associated HIV-1 RNA isolated from total CD4^+^ T cells divided in small aliquots so that they would contain only 1 HIV-1 RNA–producing cell, as previously described (46). We detected low-level U5-PBS RNA (average of 11 copies/10^6^ cells) and recovered a single U5-gag unspliced RNA sequence, which matched ZNF470i. Taken together, these results confirm the low spontaneous transcriptional activity of ZNF721i and ZNF470i in circulating cells, in vivo (Supplemental Figure 2).

Finally, we repeated the CD8-depleted PBMC stimulation with individual Gag peptides that induced responses by ELISpot and observed increased LTR copies in response to 6 out of 9 peptides (Supplemental Figure 3). Although these proviruses were defective by intact proviral DNA assay (IPDA) (data not shown), our results suggested that HIV-1–reactive CD4^+^ T cells carrying proviruses are common in this study participant.

### Differential inducibility of 2 proviruses in cells reactive to specific Gag epitopes.

**Figure 4:**
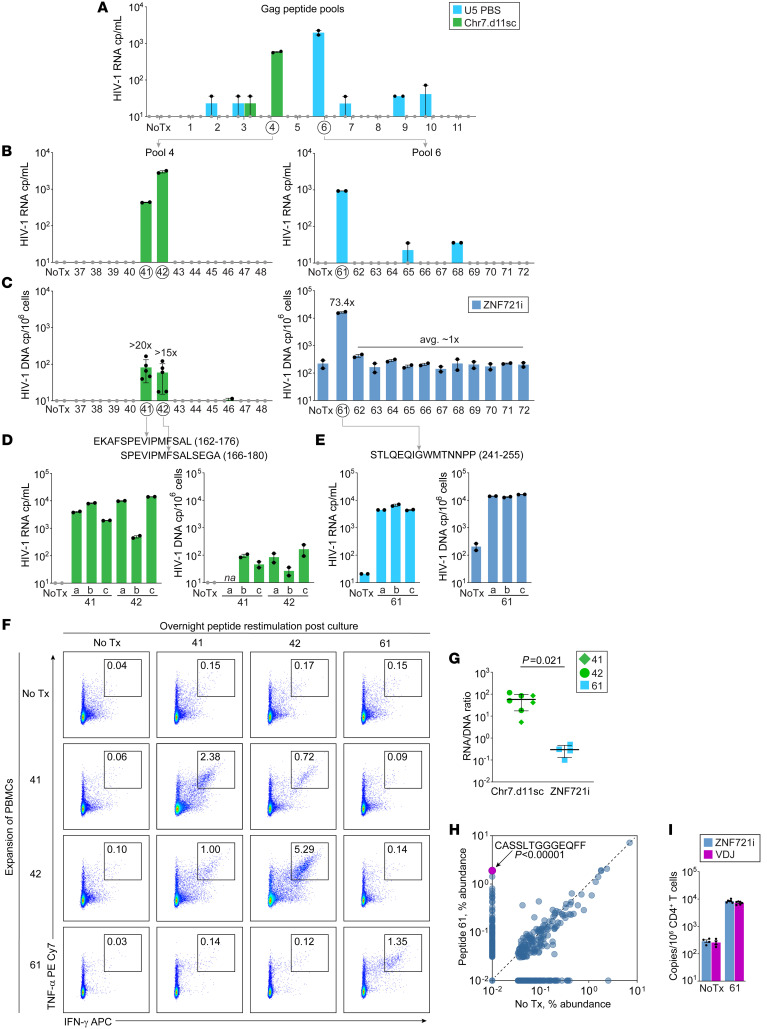
PBMC stimulation with HIV-1 Gag peptides induces proliferation of infected cells and virus production. (**A**) CD8-depleted PBMCs from day 1111 after CRT were stimulated with 11 minipools, each containing 12 Gag peptides; No Tx indicates no treatment negative control; black dots indicate technical replicates of day 9 HIV-1 RNA measurement in supernatant. (**B**) Virus production at the end of culture of CD8-depleted PBMCs from day 1142 after CRT with individual epitopes from minipools showing virus production in **A**. (**C**) Frequency of proviruses of interest by day 9 of culture; fold increase compared to no treatment is indicated above each bar; peptides with increased virus production and cell proliferation are indicated below the graph. (**D** and **E**) Validation experiments with individual peptides in triplicate; the statistical significance between peptides 41 and 42 was investigated by 2-tailed parametric *t* test. (**F**) Intracellular cytokine staining of CD4^+^ T cells restimulated with Gag peptides of interest after PBMC expansion; numbers within gates indicate the percentage of cells positive for both TNF-α and IFN-γ. (**G**) Differential inducibility between proviruses of interest upon stimulation with cognate Gag peptides; inducibility is expressed as the ratio between HIV-1 RNA copies in the supernatant and proviral copies in cells at the end of culture. (**H**) Analysis of differential abundance (%) of the top 1000 productive TCRs between no treatment and stimulation with peptide 61; the differential abundance of the clonotype carrying the ZNF721i provirus (CASSLTGGGEQFF) was evaluated by χ^2^ test. (**I**) Frequency of both provirus and VDJ rearrangement belonging to the CASSLTGGGEQFF clonotype in CD4^+^ T cells at the end of culture.

To extend our results, we sought to identify the Gag specificity of the clones carrying Chr7.d11sc and ZNF721i at the epitope level, so that we could investigate proviral inducibility upon encounter with their cognate antigen (Figure 4). As a readout for T cell response to pooled or individual Gag peptides, we used both virus production in the supernatant and infected-cell proliferation (Figure 4, A–E; see Methods). With this approach, we found that the clone harboring Chr7.d11sc is reactive to 2 overlapping peptides, EKAFSPEVIPMFSAL (peptide 41, Gag 162–176) and SPEVIPMFSALSEGA (peptide 42, Gag 166–180), while cells harboring ZNF721i respond to STLQEQIGWMTNNPP (peptide 61, Gag 241–255). Conversely, ZNF470i did not proliferate or produce virus upon Gag pool stimulation (Supplemental Figure 4). Since the Chr7.d11sc provirus is rare and can be detected only in cells after stimulation with peptides 41 and 42, the precise fold increase cannot be quantified; however, we can estimate a minimum of 15- to 20-fold increase using the limit of detection in untreated cells (4 copies/10^6^ cells). In subsequent experiments we isolated CD4^+^ T cells at the end of culture, which confirmed that in the no-treatment condition, the frequency of Chr7.d11sc is 2.4 ± 3.3 copies/10^6^ CD4^+^ T cells. Upon stimulation with peptide 61, the frequency of ZNF721i showed a 73-fold increase relative to no treatment. The stimulation with individual cognate peptides is reproducible, as shown by comparable virus production and cell expansion in triplicate experiments (Figure 4, D and E, and Supplemental Figure 5). In addition, we confirmed that peptides 41, 42, and 61 are recognized by CD4^+^ T cells, based on the increase in cells positive for intracellular TNF-α and IFN-γ after 9 days of culture (Figure 4F). Interestingly, peptides 41 and 61 are also recognized by CD8^+^ T cells (Supplemental Figure 6); indeed, they overlap with well-characterized epitopes binding to HLA-B*57 (KAFSPEVIPMF and TSTLQEQIGW, respectively) (47).

We hypothesized that the striking expansion of ZNF721i in vivo and its extensive proliferation ex vivo upon antigen recognition are favored by its chromosomal location, allowing proliferation to occur without the cytopathic effects of viral replication. To test this hypothesis, we estimated the proviral inducibility in vitro of ZNF721i and Chr7.d11sc by dividing the copies of viral RNA in the supernatant by the provirus frequency in cells at the end of culture (Figure 4G). We found that the inducibility in vitro of ZNF721i was almost 200-fold lower (average 57 versus 0.3, *P* = 0.021). Lastly, we used bulk TCRβ sequencing, VDJ-specific dPCR, and combinatorial statistics to identify CASSLTGGGEQFF as the putative CDR3β sequence of the clonotype carrying ZNF721i (Figure 4, H and I). CASSLTGGGEQFF had a relative abundance of 0.01% in untreated cells, but was among the most abundant clonotypes upon stimulation with peptide 61 (1.8%, test for differential abundance *P* < 1 × 10^–5^). Based on provirus- and VDJ-specific quantification in PBMCs stimulated with peptide 61 and the unstimulated control, we estimated that almost 100% of the clonotype carries ZNF721i, suggesting that most of its clonal selection occurred after HIV-1 integration (Figure 4I and Supplemental Figure 7) (20).

### Proliferation of cells harboring ZNF721i is not affected by autologous CD8^+^ T cells.

**Figure 5:**
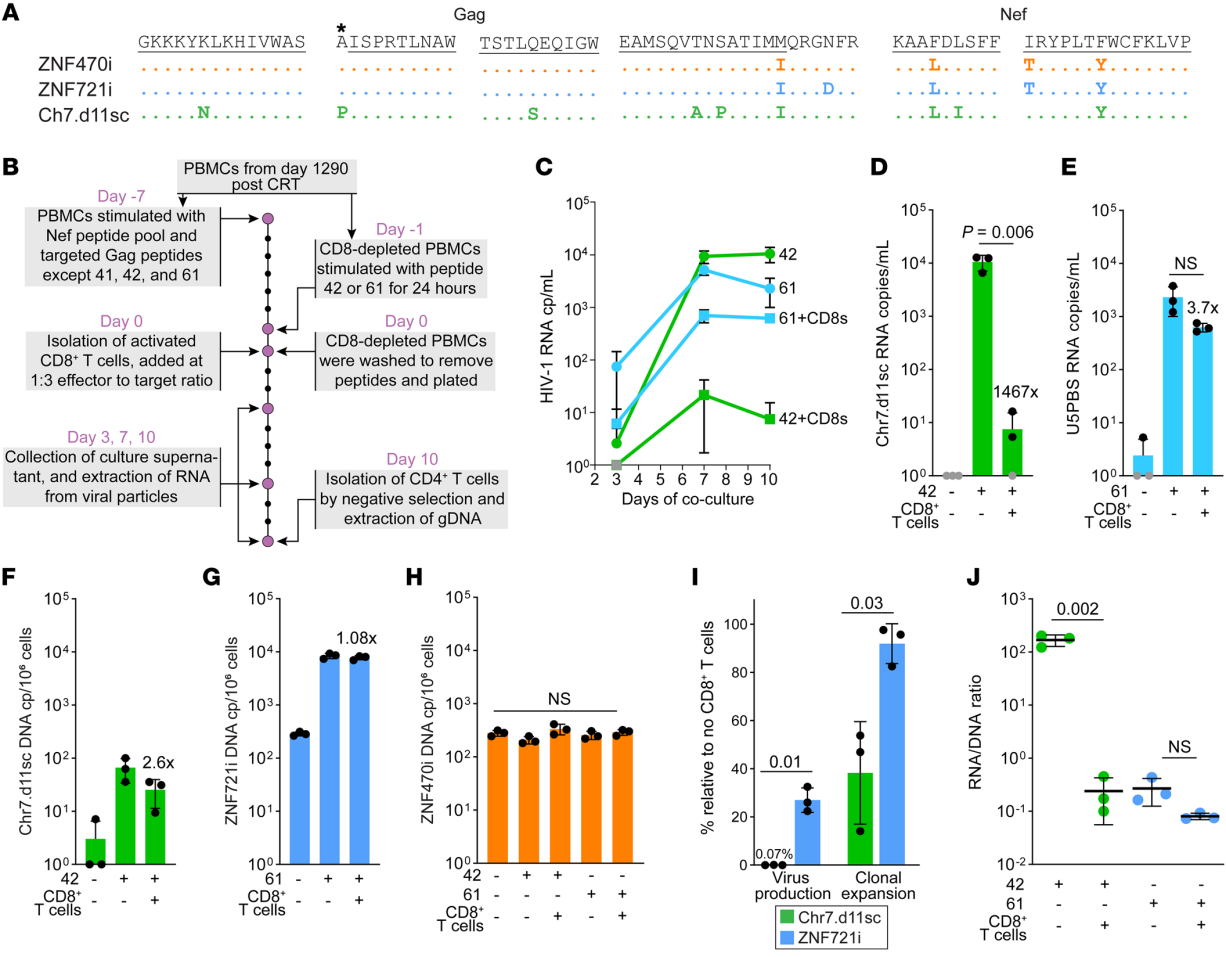
Differential impact of autologous CD8^+^ T cells on virus production and cell proliferation. (**A**) Analysis of escape mutations in Gag and Nef in the proviruses of interest; black lines indicate sequences recognized by CD8^+^ T cells in the context of HLA-B*57; asterisk marks an amino acid residue involved in epitope processing that, if mutated, contributes to escape. Full-length protein sequences are shown in Supplemental Figures 8 and 9. (**B**) Experimental design to test the impact of CD8^+^ T cells on virus production and proliferation of infected cells; aliquots of PBMCs from day 1290 after CRT were used to generate activated CD8^+^ T cells and, separately, stimulated CD8-depleted PBMCs with peptide 42 or 61. (**C**) HIV-1 RNA from culture supernatant on days 3, 7, and 10; symbols indicate the average of 3 replicate wells, error bars indicate SD; fold reduction between conditions without and with CD8^+^ T cells is indicated. (**D** and **E**) HIV-1 RNA on day 10 across different conditions; black symbols represent replicate wells; values below the limit of detection are indicated in gray; bars show average and SD. (**F**–**H**) Quantification of proviruses of interest measured by dPCR targeting either the 11-nt deletion (Chr7.d11sc) or integration sites (ZNF721i and ZNF470i) expressed as copies per million CD4^+^ T cells. (**I**) Impact of immune recognition on virus production and proliferation of infected cells, expressed as percentage relative to wells without CD8^+^ T cells. (**J**) RNA-to-DNA ratios of Chr7.d11sc and ZNF721i upon stimulation with cognate peptides with or without CD8^+^ T cells. The statistical differences between conditions were evaluated by 2-tailed parametric *t* test.

Clonal expansion of infected clones is the result of opposing forces, such as the frequency of cell stimulation, proliferation rate, and cell death due to viral cytopathic effects. However, immune recognition by cytotoxic cells imposes a major selective force in shaping reservoir dynamics and composition, especially in ECs, resulting in the observed differential genomic distribution of intact proviruses (18, 27). Thus, we sought to determine whether lower proviral inducibility allows infected clones to escape immune recognition. Analysis of Gag and Nef amino acid sequences revealed that Chr7.d11sc had more escape mutations in known B*57-restricted epitopes than ZNF470i and ZNF721i (Figure 5A and Supplemental Figures 8 and 9), suggesting a lower susceptibility to CD8-mediated killing for cells carrying Chr7.d11sc. PBMCs were stimulated for 7 days with overlapping peptides covering the entire Nef protein and with selected Gag peptides that showed response by ELISpot (see Methods). Of note, we excluded peptides 41, 42, and 61 to avoid stimulation of infected cells of interest (Figure 5B). In parallel, CD8-depleted PBMCs were stimulated with either peptide 42 or 61 for 24 hours; peptides were then removed, and cells were plated with stimulated CD8^+^ T cells at a 1:3 effector-to-target ratio and cultured for 10 days. Virus production, monitored from day 3 to day 10, was markedly reduced by CD8^+^ T cells; however, Chr7.d11sc was significantly more affected than ZNF721i (1467- vs. 3.7-fold reduction in HIV-1 RNA copies/mL on day 10, *P* = 0.02; Figure 5, C–E). Although CD8^+^ T cells had a lesser effect on cell proliferation, likely reflecting that most infected cells divide without the expression of viral antigens (13, 48), cells carrying Chr7.d11sc proliferated significantly less than the ZNF721i clone (Figure 5, F, G, and I). Of note, cells harboring the ZNF470i provirus were not affected by CD8^+^ T cells (Figure 5H), indicating that in this context CTLs specifically recognized and killed only peptide-stimulated infected cells, rather than via nonspecific effector molecule production and noncytolytic effects (49–51). In the presence of activated CD8^+^ T cells, cells harboring Chr7.d11sc decreased to about 40% of the cells in the control arm, while the ZNF721i provirus was barely affected (95% of cells without CD8s, *P* = 0.03; Figure 5I). Coculture with CD8^+^ T cells had a strikingly distinct effect on virus production and cell proliferation, suggesting that only virus-producing cells were eliminated. Moreover, the amount of virus produced on a per-cell level may affect the efficiency of immune recognition and the HIV RNA levels at the end of culture. Indeed, in our experiments we observed that the estimated inducibility of Chr7.d11sc was significantly reduced by CD8^+^ T cells (average RNA/DNA ratio 169 versus 0.24, *P* = 0.002; Figure 5J), while ZNF721i was only modestly affected (0.27 versus 0.08, *P* = NS). Together, these results demonstrate that the lower inducibility of the ZNF721i provirus allowed escape from CTL recognition by proliferating upon T cell activation, with negligible expression of viral antigens, which can explain its marked clonal expansion observed in vivo after CRT and immunotherapy.

## Discussion

The activation of latently infected CD4^+^ T cells with potent mitogens results in viral transcription and the reversal of latency in many clones (52). However, this process is stochastic and there is mounting evidence that following activation, some infected T cell clones can proliferate without producing virus (13, 14, 48). This process has major implications for the persistence of infected CD4^+^ T cell clones in the presence of HIV-1–specific CD8^+^ T cells (53–55).

In this case report, we characterized clonal dynamics before, during, and after chemoradiation and immunotherapy in an EC with metastatic lung cancer. The contraction of individual clones is remarkable, as chemotherapy and immunotherapy generally did not affect the size of the latent reservoir in prior studies (26, 56, 57). The same pattern of a modest but transient reduction in intact proviral DNA was recently described in kidney transplant patients receiving induction immunosuppression (33). Our results extend these findings by tracking individual clones with integration site analysis. Three clones in this study are especially interesting. The large clone, which contains replication-competent ZNF470i provirus, was stable during treatment. In contrast, the cells carrying the ZNF721i provirus, which recognizes the Gag peptide STLQEQIGWMTNNPP (amino acids 241–255), underwent a 70-fold expansion after chemotherapy was completed. This may explain why there was only a modest decline in intact proviral DNA despite the marked reduction in the number of total clones after chemoradiation. The third clone, Chr7.d11sc, contains a nearly intact provirus and recognizes the overlapping Gag peptides EKAFSPEVIPMFSAL (amino acids 162–176) and SPEVIPMFSALSEGA (amino acids 166–180). To the best of our knowledge, this is the first study to identify the exact epitope recognized by infected T cell clones. This is important, as epitope identification could enable the specific activation of latently infected T cells as part of HIV-1 cure strategies. While the peptides triggered both viral transcription and proliferation of both the ZNF721i and Chr7.d11sc clones, the degree of viral transcription was significantly lower for the ZNF721i provirus. This difference in viral transcription between the 2 clones was associated with differences in the ability of autologous CD8^+^ T cells to inhibit clonal expansion. While the presence of CD8^+^ T cells resulted in a 60% inhibition of proliferation of cells with the Chr7.d11sc provirus, CD8^+^ T cells had no effect on the proliferation of the clone carrying ZNF721i. This difference is particularly interesting given the fact that the Chr7.d11sc provirus has accumulated several escape mutations, while the ZNF721i provirus has no escape mutations in Gag and Nef epitopes. The presence of escape mutations in Chr7.d11sc suggests that some of the reservoir cells in ES24 have been subjected to CD8^+^ T cell selective pressure in vivo. While these mutations are rare in proviruses from ECs, they have been described, and virologic control may be maintained by de novo CD8^+^ T cell responses that can inhibit replication of autologous escape mutants (58–61).

The 60% reduction of the Chr7.11sc provirus may be an underestimation of the ability of CD8^+^ T cells to inhibit the expansion of virus-producing cells given the stochastic nature of latency reversal. In a prior study that subjected CD4^+^ T cells to multiple rounds of potent activation, a mean of just 60% of the infected cells that eventually produced virus did so after the first round of activation (36). Thus, it is likely that only a subset of cells of the Chr7.d11sc provirus produced virus in response to 24 hours of stimulation with cognate peptide. The 3-log reduction in Chr7.d11sc RNA in the presence of CD8^+^ T cells suggests that most of the cells that produced virus were eliminated, and the remaining cells were not targeted because they did not express viral proteins. The ZNF721i provirus is significantly less inducible than the Chr7.d11sc provirus, which may contribute to immune evasion and explain the rapid expansion of cells with the ZNF721i provirus in contrast to the Chr7.d11sc provirus, which is present at a much lower frequency in vivo.

Our study is limited by the fact that it involves a single patient who is an EC. Even though this case may not be representative of most people without spontaneous HIV-1 control, a progressive enrichment of intact proviruses in ZNF genes has been observed in PLWH on long-term ART. It is also possible that the process of clonal expansion was affected by his malignancy. However, we describe the effects of chemoradiation and immunotherapy on individual clones and study the effects of T cell activation on infected clones using a physiologic system with individual cognate peptides. In addition, our findings can be extended to larger cohorts of ECs, whose HIV-1–reactive CD4^+^ clones can be easily detected (62). The highly dominant ZNF721i provirus in Gag-reactive CD4^+^ clones, found in this participant, has not been described previously to our knowledge. However, the presence of HIV-1–reactive clones, albeit rarer among all infected cells, is generalizable to both ECs and normal progressors on long-term ART (20, 40, 63). Our findings suggest that the selective recognition and elimination of transcriptionally active proviruses like Chr7.d11sc by the immune system could explain the predominance of clonally expanded proviruses integrated into transcriptionally repressed sites in HIV-1 controllers and people on long-term ART (18). The experimental evidence provided here further supports recent reports of genotypic and phenotypic signatures of intact proviruses, results of a dynamic proviral landscape shaped over time by the immune system (64).

However, additional work is needed to understand whether specific host and proviral factors contribute to the establishment and maintenance, the so-called “deeper latency” of some intact proviruses in ZNF genes. Whether rare proviruses are merely caught in between the evolutionary arms race against transposable elements or specifically targeted by repressive epigenetic mediators is yet to be determined (65, 66). Moreover, our data also suggest that strategies that use agents like IL-15, which induce proliferation to augment effector responses, may paradoxically increase the size of the reservoir by causing expansion in the absence of viral expression of proviruses like ZNF721i that can partially evade the immune system (67). Finally, it should be noted that viral particle production does occur even from proviruses like ZNF721i, and thus immune recognition and elimination of productively infected cells would still be essential for elite control and for preventing viral rebound in most people on ART.

## Methods

### Study participant.

The study participant, a 66-year-old African American male, has been anonymized as ES24. He was diagnosed HIV-1–positive in 2009. Seven total longitudinal time points have been collected for this study. The participant’s PBMCs were obtained from whole blood by Ficoll-based density separation, and CD4^+^ T cells were isolated by magnetic bead–based negative selection (Miltenyi Biotec). Activated lymphoblasts and irradiated PBMCs from HIV-1–negative individuals were used in the qVOA assay.

### IPDA.

The IPDA was performed as previously described (68) on CD4^+^ T cells, using the QX200 Droplet Digital PCR System (Bio-Rad). The assay interrogates 2 specific regions of the HIV-1 genome to genetically discriminate between defective and intact proviral DNA.

### qVOA.

The qVOA was performed as previously described (58, 69) in isolated CD4^+^ T cells. Following identification of p24^+^ wells, supernatants were collected and extracted to sequence the virion-associated HIV-1 RNA in either the *nef* or U5-*gag* region. (15).

### Integration site analysis.

Integration site analysis was performed with 2 different approaches. Linker-mediated PCR was performed on genomic DNA from bulk CD4^+^ T cells following controlled sonication, as previously described (70). PCR products were purified, pooled, and sequenced on an Illumina MiSeq. Integration sites were determined from the sequence data using the INSPIIRED pipeline (https://github.com/BushmanLab/). Conversely, the Lenti-X Integration Site Analysis Kit (Clontech) was used to characterize the integration sites from limiting-diluted genomic DNA (gDNA) subjected to whole genome amplification, using the Advance Single Cell Repli-G Kit (QIAGEN), as previously described (35). PCR products obtained with this approach were sequenced by Sanger sequencing (Azenta).

### Provirus sequencing.

gDNA was isolated accordingly to the number of cells. For samples with fewer than 1 million cells, DNA was extracted following Wiegand et al. (46); otherwise, the QIAamp DNA Mini Kit (QIAGEN) was used. Following limiting dilution, proviral sequences of ZNF470i and ZNF721i were recovered by FLIPS (71) and MIPseq (35), respectively. Due to the limited presence of Chr7.d11sc, its viral sequence was recovered from virion-associated HIV-1 RNA in the supernatant, obtained from CD8-depleted PBMC stimulation experiments (see below). If primers commonly used for full-length proviral sequencing failed due to mismatches, specific primers were designed. Finally, to reconstruct the full proviral sequence, primers annealing in the human genome flanking the integration site were used to recover the LTR regions. All the primers used in this study are detailed in Supplemental Table 2.

### Total LTR copies.

To quantify all LTR copies we used primers and probe targeting the R-U5 junction, as previously described (38, 72). In brief, gDNA from total CD4^+^ T cells undergoes controlled physical shearing so that the LTRs from the same provirus are not in linkage and are captured in separate partitions. The primers and probe anneal to the R-U5 junction, so that both LTRs are quantified regardless of 5′ or 3′ end. dPCR reactions were run using the QIAcuity One Digital PCR System (QIAGEN) with an initial denaturation step of 95°C for 2 minutes, followed by 45 cycles each including 95°C for 15 seconds and 58°C for 30 seconds. This assay can be run in a multiplex reaction with the integration site PCR described below. Oligonucleotide sequences are described in Supplemental Table 2.

### Quantification of proviruses of interest by dPCR.

Specific assays to quantify ZNF470i and ZNF721i were designed based on the host-U3 junction, with the fluorescently labeled probe annealing across the site of HIV integration, as previously described (20). In addition, exploiting the 11-nucleotide deletion present in the primer binding site of Chr7.d11sc (HIV-1 group M subtype B, HXB2 positions 660–670), we designed an assay based on competition probes to discriminate the Chr7.d11sc virus from other variants. To confirm that the virus produced upon stimulation with peptide 61 was indeed from ZNF721i provirus, we performed single genome sequencing on the supernatant (Supplemental Figure 5). Copies of targets of interests were normalized based on cell genome equivalents screened, calculated by RPP30, as previously described (68). Primers and probes are detailed in Supplemental Table 2. dPCR reactions were run using the QIAcuity One Digital PCR System, with an initial denaturation step of 95°C for 2 minutes followed by 45 cycles of 95°C for 15 seconds and 58°C for 30 seconds.

### Clonal expansion assay.

PBMCs were obtained from blood via a standard Ficoll procedure. The cells were subjected to 2 rounds of CD8^+^ T cell depletion using negative selection beads (Miltenyi Biotec). CD8-depleted PBMCs were then cultured in RPMI (Gibco) with 10% fetal calf serum (Gibco) and 10 units/mL of IL-2 at a concentration of 1 million cells/mL. The antiretroviral drugs raltegravir and efavirenz were obtained from the NIH HIV Reagent Program. Four million cells were cultured in each well of a 12-well plate and stimulated with overlapping Gag peptides from the NIH AIDS Reagent Program at a concentration of 10 μg/mL. To identify the individual Gag peptides that induced clonal expansion, CD8-depleted PBMCs were stimulated with 11 different pools containing 10–13 peptides each. Each individual peptide from the 2 pools that resulted in virus production was then tested individually at a concentration of 10 μg/mL.

### RNA extraction and cDNA synthesis.

Culture supernatants were spun at 3500*g* for 15 minutes at 4°C and transferred to clean tubes for an additional centrifugation step at 21,000*g* for 2 hours at 4°C. Viral pellets underwent RNA extraction, as previously descried (73). RNA was immediately used for reverse transcription using Super Script III with a participant-specific primer located in *gag*. The cDNA synthesis reaction was at 55°C for 50 minutes, followed by an inactivation step at 85°C for 5 minutes. cDNA was quantified by a dPCR assay targeting the U5-PBS region, or used for single genome sequencing, as described above. To quantify and sequence cell-associated RNA from peripheral blood CD4^+^ T cells, we isolated total RNA and gDNA from small aliquots of cells (700,000 cells), as previously described (46). HIV-1 U5-PBS and Chr7.d11sc RNA were quantified as described above, and normalized to 1 million cell equivalents by RPP30 copies from gDNA.

### TCR sequencing and quantification of VDJ rearrangements of interest.

gDNA from cultured cells was subjected to TCRβ sequencing, as previously described (74). To measure CASSLTGGGEQFF, we designed a dPCR assay with primers flanking the VDJ rearrangement of the TCR β chain, as previously described (20). To increase specificity and avoid a polymeric region of guanosines, we designed 2 different probes annealing across the VD and DJ junctions, fluorescently labeled with fluorescein amidites (FAM) and hexachlorofluorescein (HEX), respectively. Only double-positive partitions were used to quantify the clonotype of interest (Supplemental Figure 4 and Supplemental Table 2). To confirm that the CASSLTGGGEQFF CDR3 belonged to the cells carrying ZNF721i, we distributed 800 CD8-depleted PBMCs over 96-well plates. After whole genome amplification, we identified wells positive for any provirus, ZNF721i, and CASSLTGGGEQFF by dPCR. We used combinatorial statistics to calculate the probability that ZNF721i and CASSLTGGGEQFF would colocalize in the same wells by chance, as previously described (20) (see Supplemental Figure 4).

### Intracellular cytokine assay.

One million PBMCs were cultured in each well of a round-bottom 96-well plate overnight in 200 μL of RPMI with 10% fetal calf serum with antibodies against CD28 and CD49d (2 μg/mL each; clone L293 and L25, respectively, BD Biosciences) in the presence of 1 μL/mL of Golgi Plug and Golgi Stop (BD Biosciences). The cells were then washed and stained with live/dead stain (Zombie NIR, BioLegend) and antibodies against CD3, CD4, and CD8 (Pacific Blue, UCHT1, 558117; PerCP/Cyanine 5.5, RPA-T4, 300530 and Brilliant Violet 605, RPA-T8, 301040, respectively; all BioLegend). The cells were then fixed and permeabilized with BD Cytofix/Cytoperm (BD Biosciences) and stained with antibodies against IFN-γ, TNF-α, and IL-2 (APC, B27, 506510; PE-Cy7, Mab11, 557647 and PE, MQ1-17H12, 500307, respectively; all BioLegend). The cells were then read on an LSR Fortessa (BD Biosciences). Data were analyzed using FlowJo 10.0.8 software (FlowJo, LLC).

### CD8^+^ T cell assay.

To induce potent cytotoxic function, CD8^+^ T cells were first stimulated with peptides for 7 days (75, 76). On day –7, PBMCs were stimulated with overlapping Nef peptides covering the entire protein and 10 Gag peptides the study participant had previously responded to, including Gag 24–39 (GKKKYKLKHIVWASR), Gag 93–107 (EVKDTKEALEKIEEE), Gag 141–155 (QMVHQAISPRTLNAW), Gag 269–283 (GLNKIVRMYSPTSIL), Gag 297–311 (VDRFYKTLRAEQASQ), Gag 333–347 (ILKALGPAATLEEMM), Gag 365–379 (EAMSQVTNSATIMMQ), Gag 433–447 (FLGKIWPSHKGRPGN), Gag 453–467 (PEPTAPPEESFRFGE), and Gag 483–497 (KELYPLASLRSLFGN) in R10 media with 10 units/mL of IL-2, efavirenz, and raltegravir. Gag peptides 41, 42, and 61 were all excluded so as not to activate and expand T cells specific for these peptides, which could kill target cells that had residual peptides present on MHC molecules after activation. On day –1, fresh PBMCs were subjected to 2 rounds of CD8^+^ T cell depletion with Miltenyi Biotec microbeads. The cells were then stimulated for 24 hours with peptides 41 and 61 at a concentration of 10 μg/mL in R10 media with 10 units/mL of IL-2 with efavirenz and raltegravir. On day 0, the CD8-depleted PBMCs were washed to remove peptide and plated at 3 million cells per well in a 24-well plate with 3 replicates per condition. CD8^+^ T cells were isolated from the PBMCs that had been stimulated with peptides on day –7 using Miltenyi Biotec microbeads. One million CD8^+^ T cells were added to 3 million stimulated CD8-depleted PBMCs. Half of the media was removed and fresh R10 with 10 units/mL of IL-2 was added on days 3 and 7, and efavirenz and raltegravir were added on day 7. On day 10, CD4^+^ T cells were isolated by negative selection (STEMCELL Technologies) in 96-well plates. gDNA was extracted from approximately 0.5 million CD4^+^ T cells and used for downstream dPCR assays.

### Visualization of ChIP-seq data.

Data from primary CD4^+^ T cells were obtained from the ROADMAP Epigenomics program database (https://www.ncbi.nlm.nih.gov/geo/roadmap/epigenomics/; data set IDs E037, E038, E039, E040, E041, E042, and E043) and chromatin states were visualized via Epigenome Browser (77) and Epilogos (https://epilogos.altius.org/) (45).

### Statistics.

Descriptive statistics, tests for normality, 2-tailed Student’s *t* test, and 1-way ANOVA tests were used to determine statistical significance using GraphPad Prism v8.0. Fisher’s exact and χ^2^ tests were run in Excel. Combinatorial statistics for provirus and TCR co-occurrence were calculated as previously described (20, 78). A *P* value of less than 0.05 was considered significant, unless otherwise stated.

### Data availability.

Single genome sequences and full-length proviral genome sequences retrieved for this work have been deposited to GenBank (accession numbers OR496614–OR496825, OR496614–OR496828, and OR496826–OR496828) and the NCI provirus database. Integration site data are available at the NCBI Bioproject Sequence Read Archive (reference PRNJA988676) and the NCI retrovirus integration database (https://rid.ncifcrf.gov/). TCRβ sequencing data are available in the NCBI Gene Expression Omnibus database (GEO GSM7577878–GSM7577889). Supporting Data Values used for Figures 1–5 and all Supplemental Figures are available in a separate Excel file in the supplemental material. All data sets generated from this study are available from the lead contact upon request, except the private health information of the study participant. This paper does not report original code.

### Study approval.

The Johns Hopkins Institutional Review Board approved this study. The study participant provided written, informed consent before enrollment.

## Author contributions

FRS and JNB conceived the study. AKK, FD, CCT, BAW, ACC, RTV, and AGD performed experiments and analyzed data. JNB, AMR, and EPS enrolled the study participant and gathered their clinical history and samples. HER and FDB conducted integration site experiments and analysis. KNS performed TCR sequencing and analysis. FRS, FD, RTV, and JNB conducted analyses and generated figures. FRS and JNB wrote the manuscript and received feedback and final approval from all authors.

## Supplementary Material

Supplemental data

ICMJE disclosure forms

Supporting data values

## Version 1 09/12/2023

In-Press Preview

## Version 2 11/01/2023

Electronic publication
