# Supplementary material for: Proviral location affects cognate peptide–induced virus production and immune recognition of HIV-1–infected T cell clones
Source: J Clin Invest. 2023 Nov 1;133(21):e171097. doi: 10.1172/JCI171097 (PMC10617777; doi:10.1172/JCI171097)
Supplement: Supplemental data [file jci-133-171097-s176.pdf]

**Proviral location affects cognate peptide-induced virus production and immune recognition of HIV-1-infected T cell clones.**

Filippo Dragoni, Abena K. Kwaa, Caroline C. Traut, Rebecca T. Veenhuis, Bezawit A. Woldemeskel, Angelica Camilo-Contreras, Haley E. Raymond, Arbor G. Dykema, Eileen P. Scully, Amanda M. Rosecrans, Kellie N. Smith, Frederic D. Bushman, Francesco R. Simonetti, and Joel N. Blankson.

## Supplementary Materials

**Table of contents**

### Supplementary Figures

**-Figure S1.** Epigenetic signatures surrounding the proviruses of interest.
**-Figure S2.** Outgrowth ex vivo of ZNF470i and ZNF721i.
**-Figure S3.** Proliferation of HIV-1-infected cells upon stimulation with selected Gag peptides.
**-Figure S4.** Cells carrying ZNF470i are not reactive to Gag peptides.
**-Figure S5.** Analysis of U5-gag sequence from culture supernatant, related to Figures 4 and 5.
**-Figure S6.** Intracellular cytokine staining of $CD8^+$ T cells stimulated with Gag peptides 41, 42, and 61.
**-Figure S7**. Quantification of the CASSLTGGGEQFF clonotype by duplex VDJ-specific ddPCR and analysis of provirus-TCR co-occurrence.
**-Figure S8.** Gag protein sequence analysis of proviruses of interest.
**-Figure S9.** Nef protein sequence analysis of proviruses of interest.

### Supplementary Tables

**-Table S1.** Characteristics of previously published proviruses integrated into *ZNF721/ABCA11P*.
**-Table S2.** Oligos used in this study.

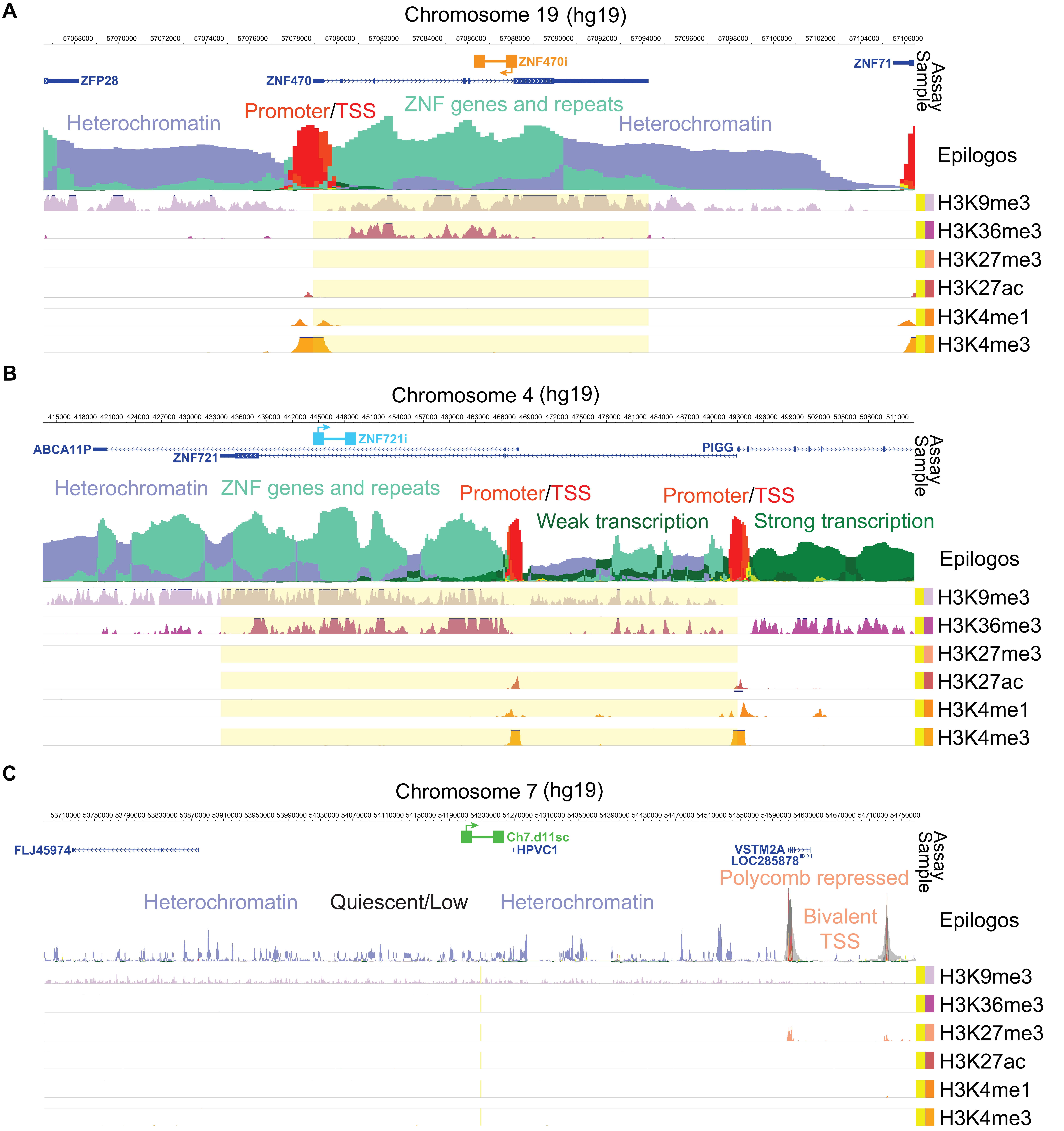


**Supplemental Figure S1. Epigenetic signatures surrounding the proviruses of interest.** CHIP-seq data from $CD4^+$ T cells available through the ROADMAP Epigenomics Program database were visualized with Epigenome broswer and Epilogos (see methods); gene tracks show genes surrounding the proviruses of interest: ZNF470i (**A**), ZNF721i (**B**), and Chr7.d11sc (**C**); Epilogos were generated based on seven datasets from primary $CD4^+$ T cells (E037, E038, E039, E040, E041, E042, E043); histone modification tracks from a representative sample (E043 Primary T helper cells from peripheral blood) are displayed below the Epilogos track.

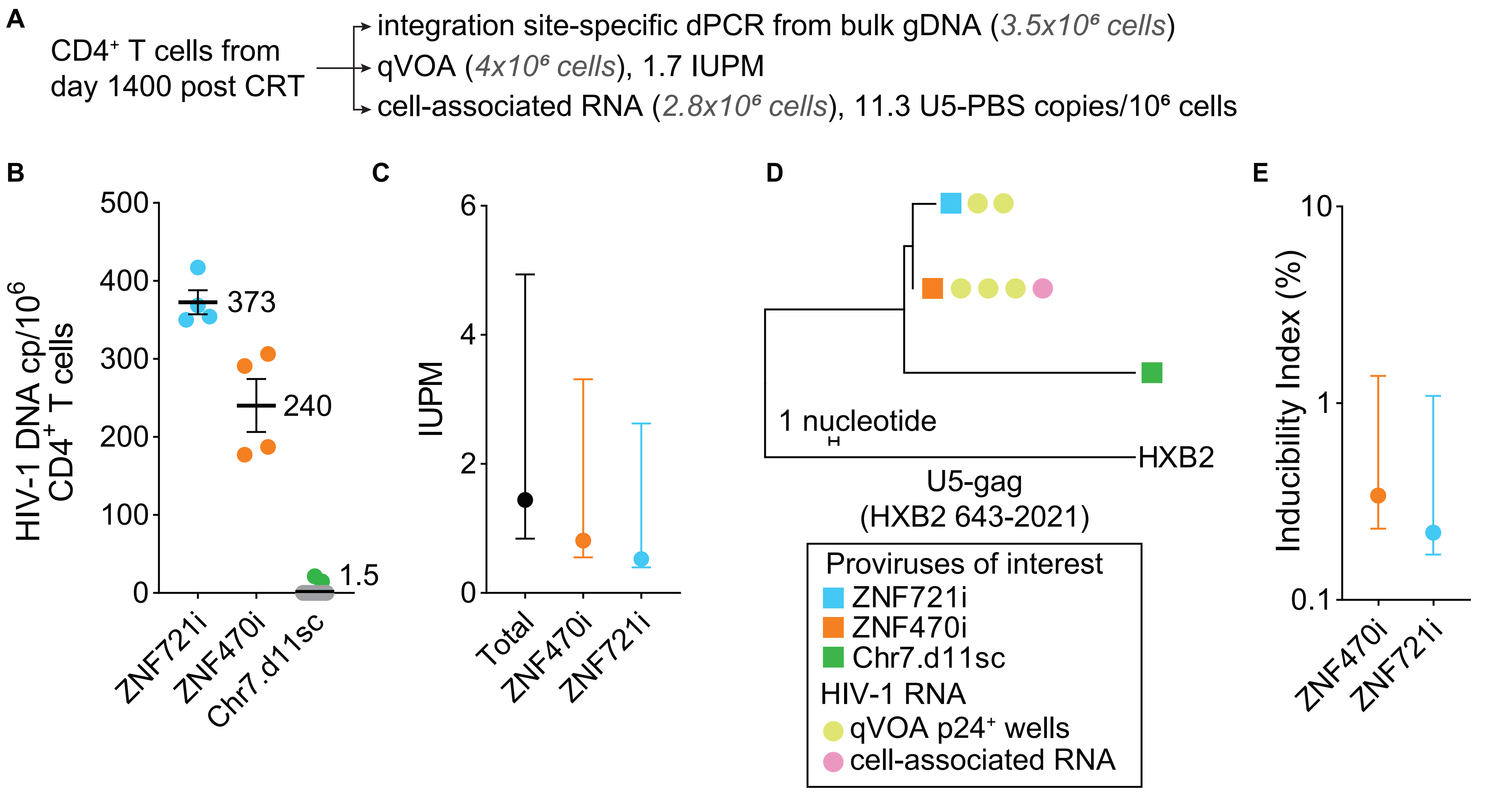


**Supplemental Figure S2. Additional reservoir characterization of CD4⁺ T cells post chemoradiation.** (**A**) Schematic of experimental design; CD4⁺ T cells were isolated from PBMCs collected from day 1400 post CRT; cells were used for quantitative viral outgrowth assay seeding 20 wells with 200,000 cells each; the remaining cells were used for the quantification of proviruses from gDNA, and isolation of cell-associated RNA from aliquots at limiting dilution for HIV-1 RNA positive cells; aliquots were screened for U5-PBS RNA and used as input for single genome sequencing; cells assayed in each method are indicated in grey. (**B**) Frequency of proviruses of interest measured by dPCR assays targeting the human-LTR junction; each symbol represents a replicate; error bars indicate mean and standard deviation; mean values are indicated in black; grey symbols indicate negative values. (**C**) Infectious units per million estimated from the qVOA; p24⁺ wells were sequenced, and IUPM were calculated for each variant matching proviruses of interest as in **D**; error bars indicate the 95% confidence intervals. (**D**) U5-gag maximum likelihood tree showing variants recovered from qVOA and cell-associated RNA, together with reference sequences of proviruses of interest. (**E**) Estimate of inducibility in vitro calculated by dividing the number of qVOA wells positive for a specific variant by the total number of its corresponding proviruses seeded in the qVOA experiment (based on their frequency as in **B**).

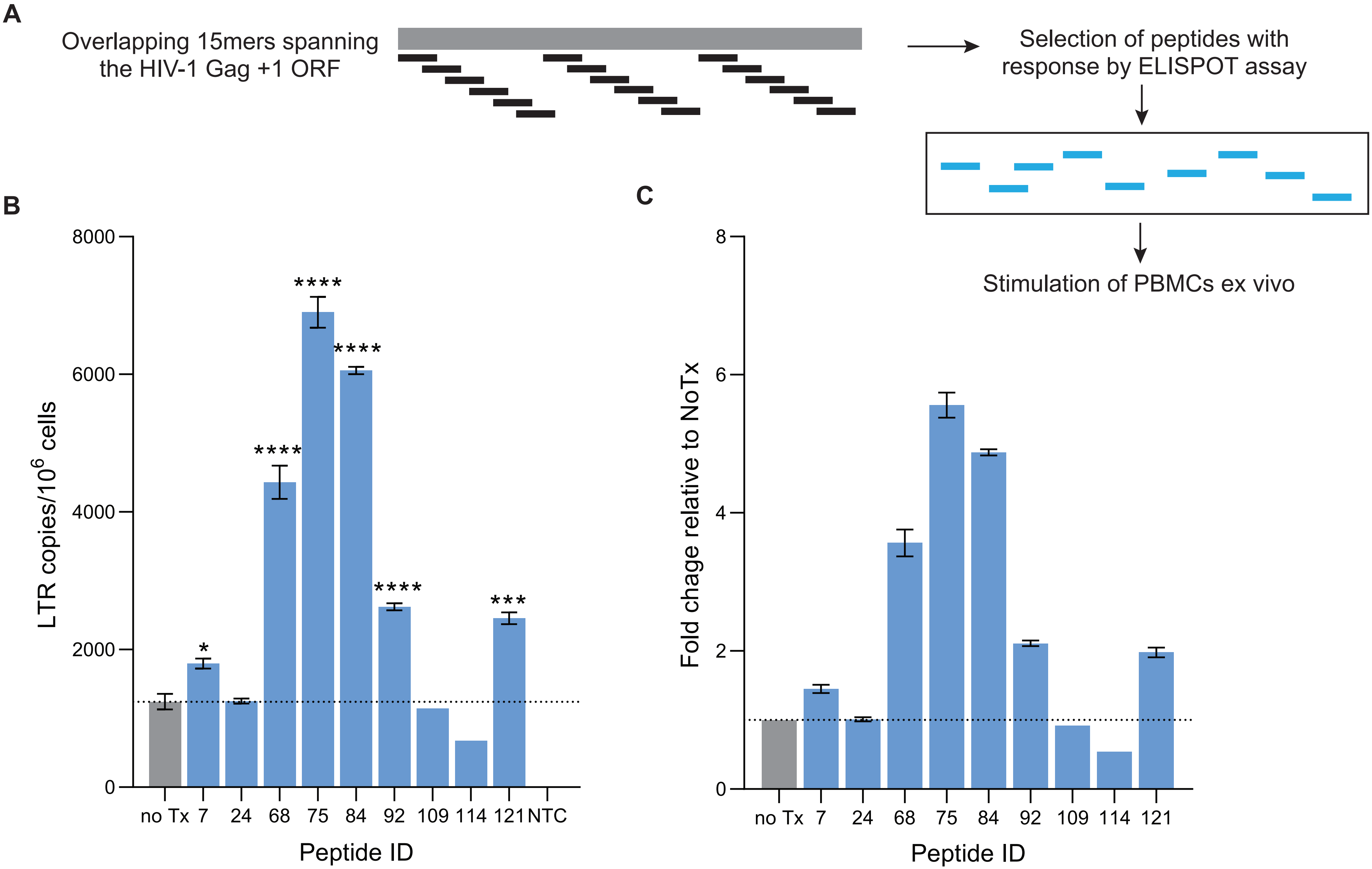


**Supplemental Figure S3. Proliferation of HIV-1-infected cells upon stimulation with selected Gag peptides.** (**A**) Schematic of experimental design; overlapping Gag peptides were selected based on response to elispot assay. (**B**) CD8-depleted PBMCs were stimulated in presence of individual peptides for 9 days; genomic DNA from total cells was used to quantify total HIV-1 LTR copies; no Tx indicate cells left untreated; NTC indicates water dPCR water control; dashed line indicates baseline HIV-1 DNA level in unstimulated cells; statistical significance was tested by one-way ANOVA; * $p<0.05$, ** $p<0.01$, *** $p<0.001$ **** $p<0.0001$. (**C**) Results expressed as fold-change relative to no treatment.

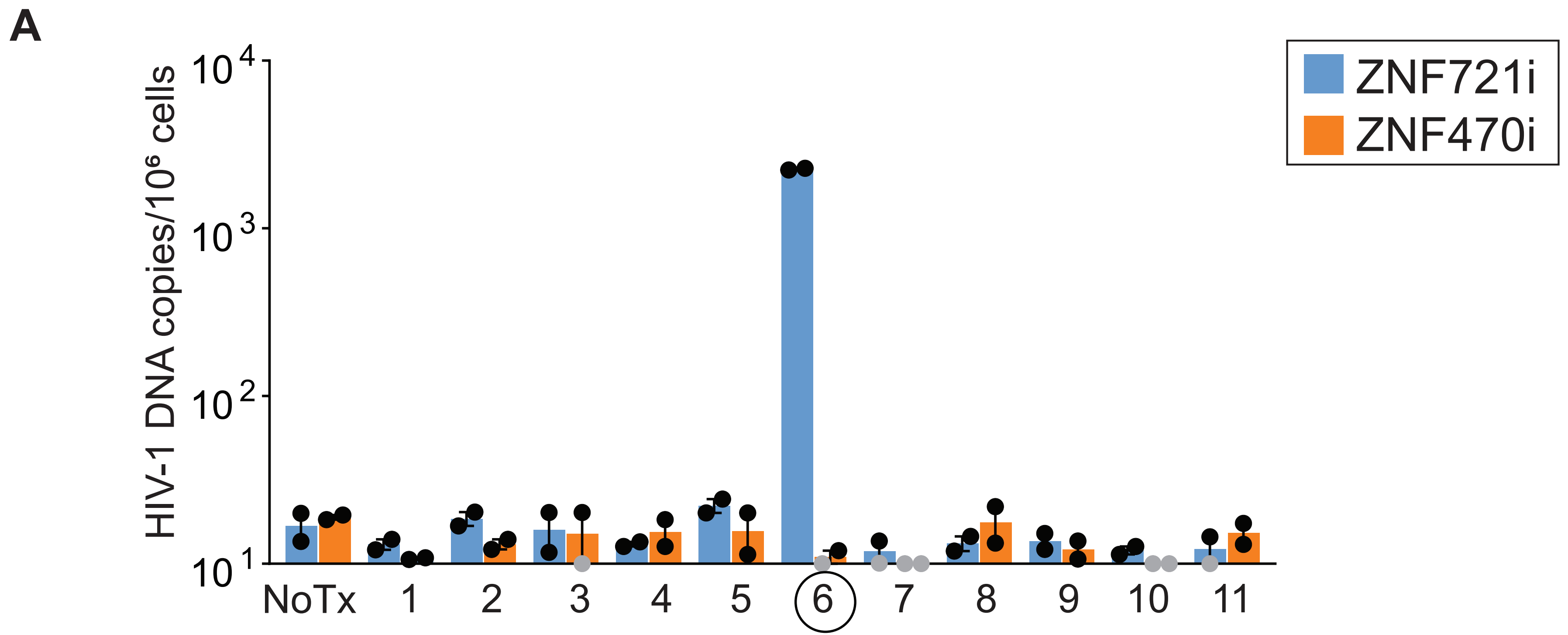


**Supplemental Figure S4. Cells carrying ZNF470i are not reactive to Gag peptides.** (**A**) CD8-depleted PBMCs were cultured for 9 days without treatment (no Tx) or upon addition of mini-pools of Gag peptides, each containing 10-11 overlapping peptides; genomic DNA was extracted from total cells at the end of culture to quantify copies of proviruses of interest harnessing the site of HIV-1 integration; each circle represents a dPCR technical replicate; grey circles indicate values below the limit of detection. Mini-pool number 6 is circled to indicate it led to a significant increase in ZNF721i copies.

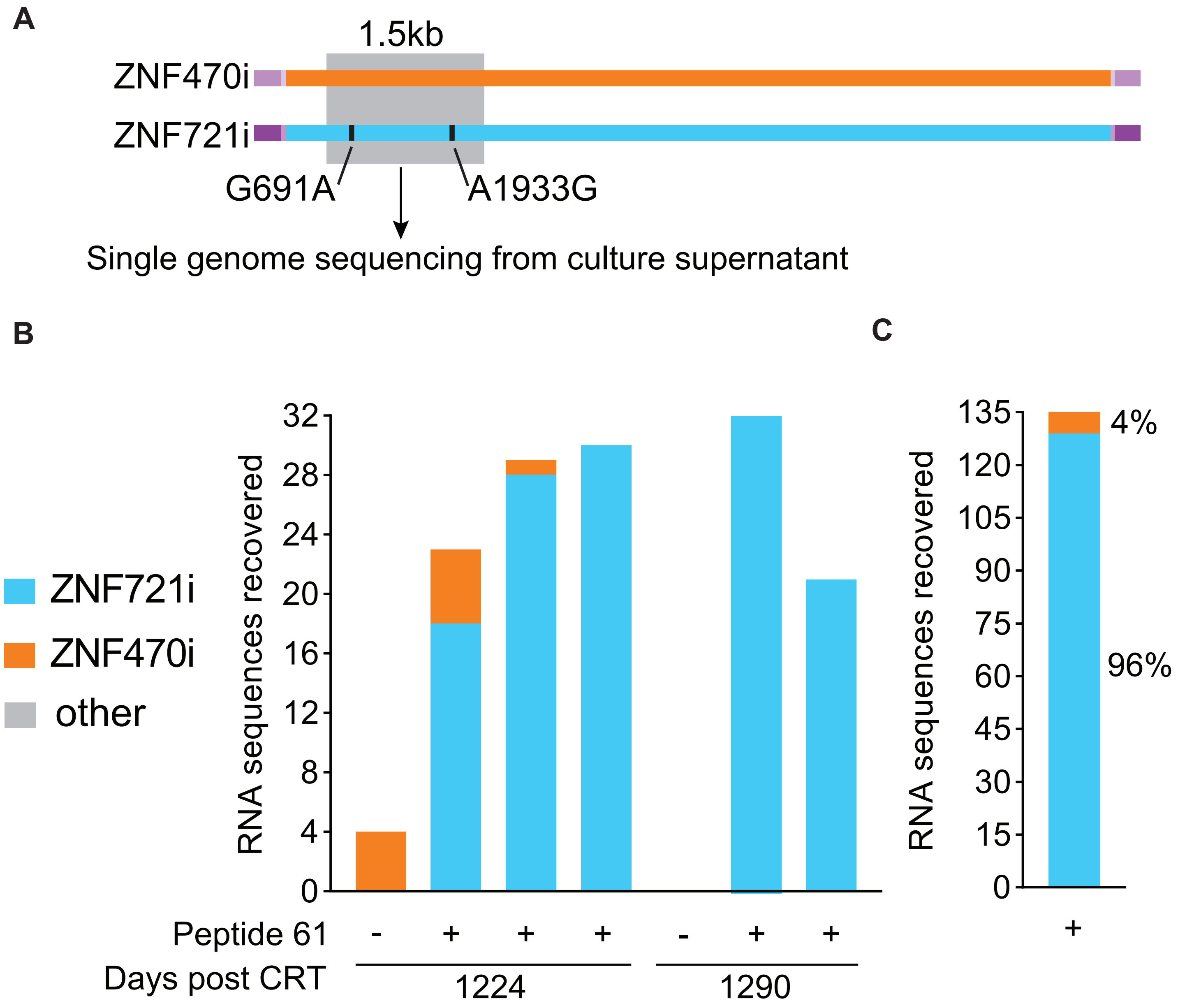


**Supplemental Figure S5. Analysis of U5-gag sequence from culture supernatant, related to Figures 4 and 5.** (**A**) Schematic of the sequencing approach to profile HIV-1 variants produced in response to CD8-depleted PBMC stimulation with peptide 61; the gray shaded area indicated the amplicon spanning between the 5'-leader and gag, which contains the two mutations that distinguish ZNF470i from ZNF721i. (**B**) Distribution of variants of interest in two control samples wihtout treatment (No Tx) and in 5 smaples treated with peptide 61. (**C**) Cumulative data from all 5 samples stimulated with peptide 61.

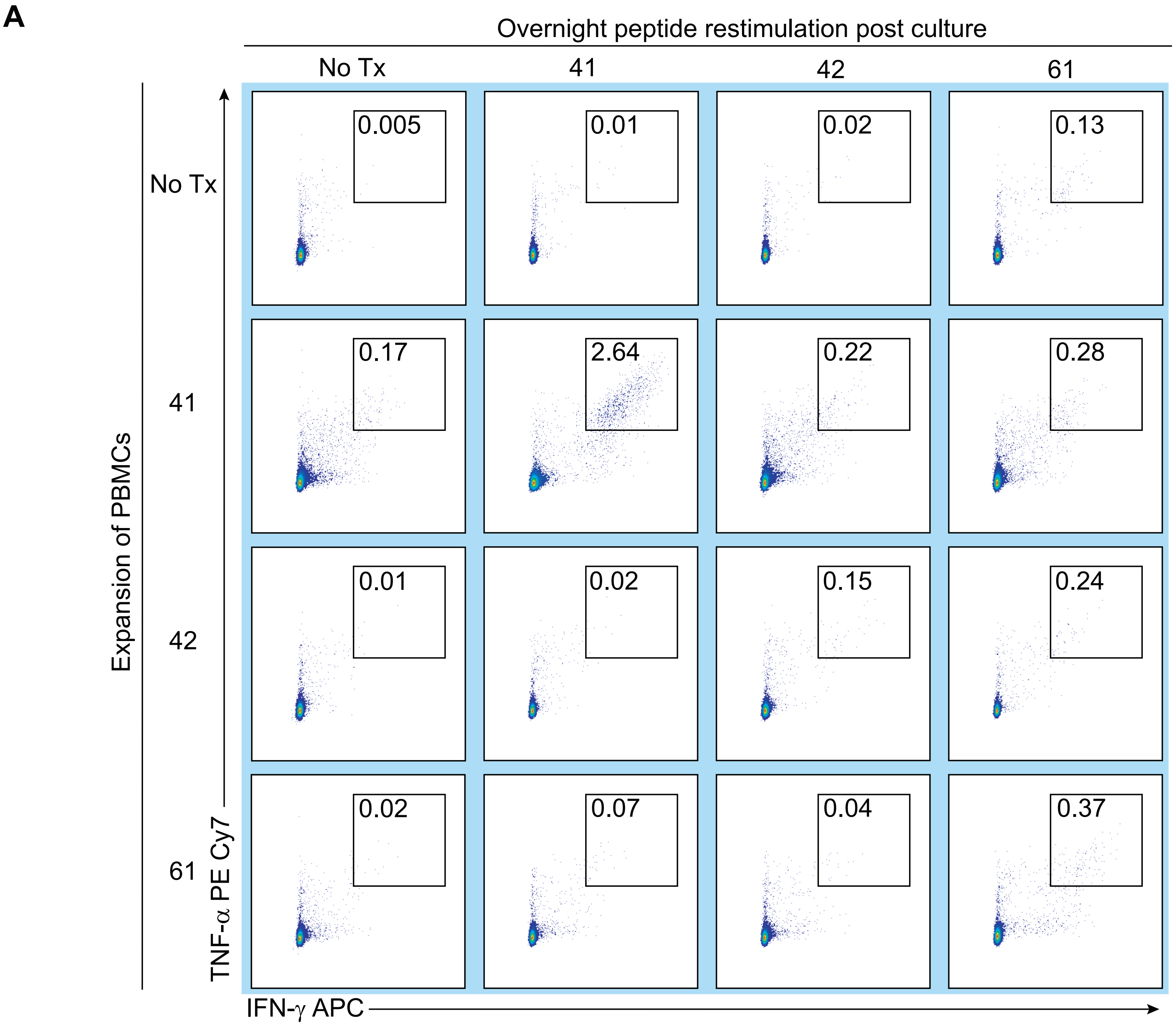


**Supplemental Figure S6. Intracellular cytokine staining of CD8⁺ T cells stimulated with Gag peptides 41, 42, and 61.** (**A**) Intracellular cytokine staining of CD8⁺ T cells re-stimulated with Gag peptides of interest after PBMC expansion; numbers within gates indicate the percentage of cells positive for both TNFα and IFNγ.

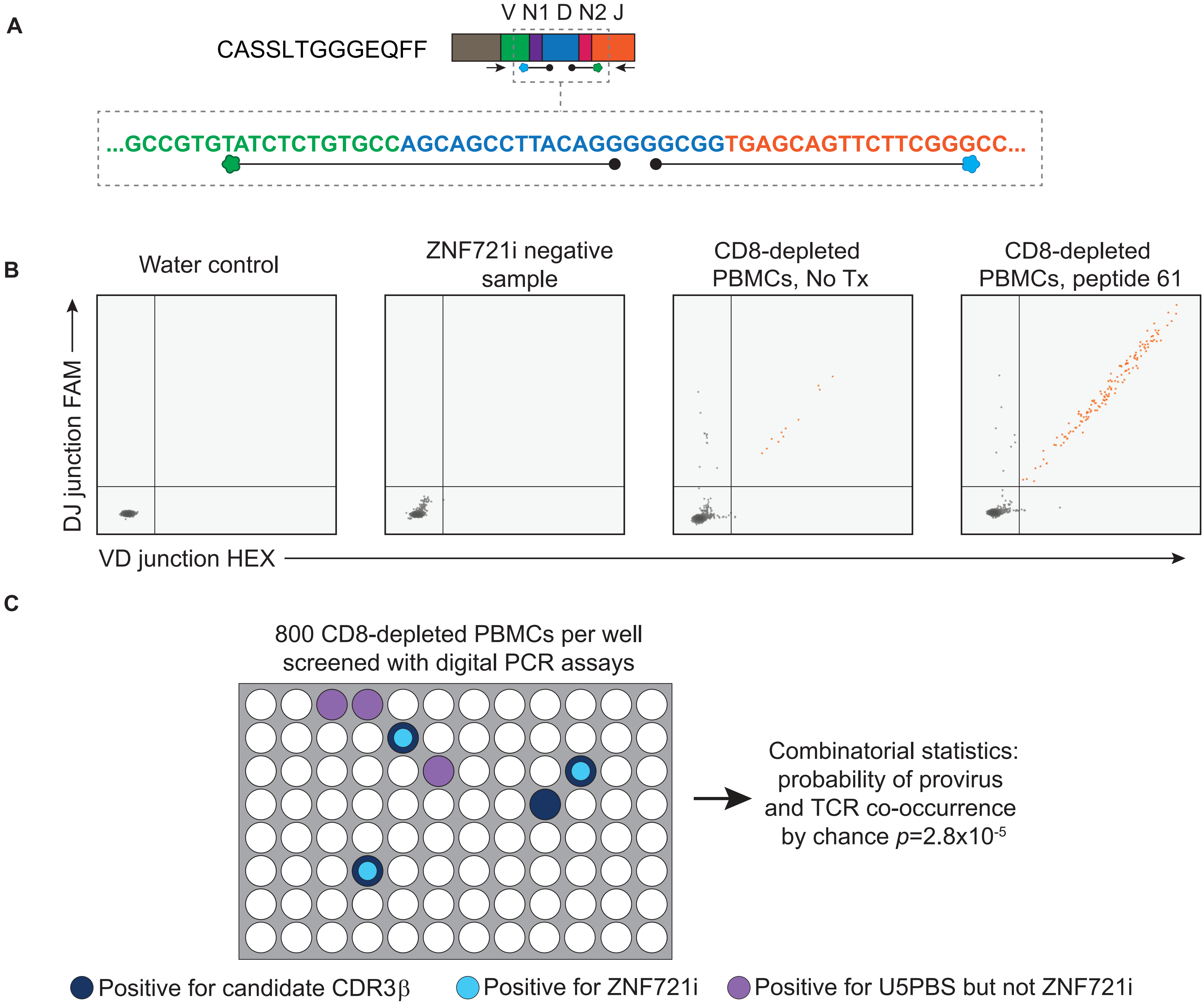


**Supplemental Figure S7. Quantification of the CASSLTGGGEQFF clonotype by duplex VDJ-specific ddPCR and analysis of provirus-TCR co-occurrence.** (**A**) Design of duplex strategy to specifically amplify the VDJ rearrangement of the CASSLTGGGEQFF CDR3β; probes were designed to anneal across the VDJ junction to exploit V, D, and J chain diversity, as well as junctional diversity (N1 and N2 nucleotides). (**B**) Representative 2D plots of droplet digital PCR reactions with water negative control, whole genome amplified DNA not containing the ZNF721i provirus, and DNA from $CD4^{+}$ T cells cultured for 10 days with no treatment, or stimulation with the cognate Gag peptide 61. (**C**) Co-occurrence analysis and conbinatorial statistics to determine whether ZNF721i and CASSLTGGGEQFF belong to the same cell clonotype.

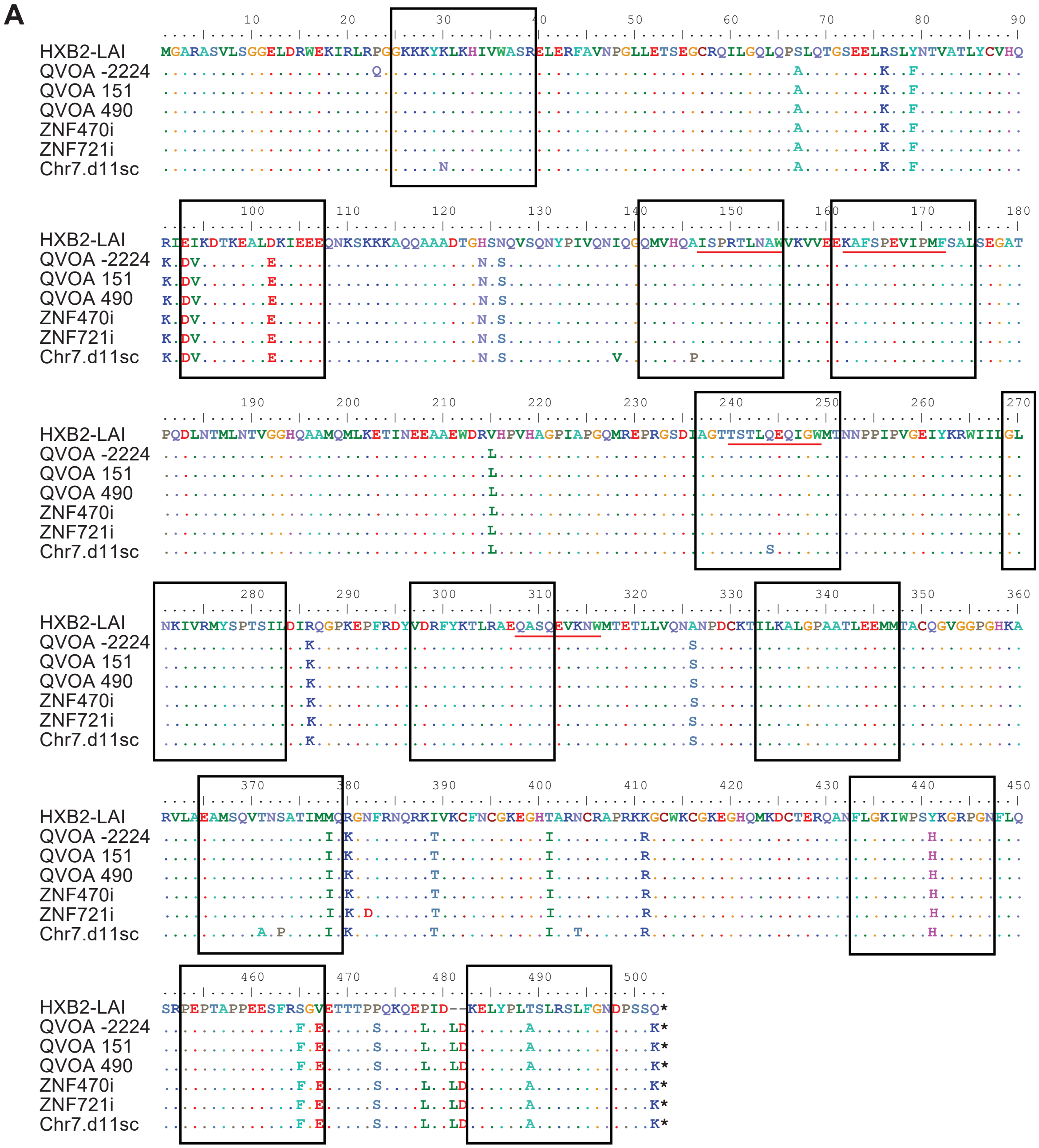


**Supplemental Figure S8. Gag protein sequence analysis of proviruses of interest.** (**A**) Gag protein sequences were codon aligned to the HXB2 reference; black boxes indicate targeted epitopes; red lines indicate HLA-B*57–restricted epitopes; numbers next to QVOA sequences indicate days from the start of chemoradiation.

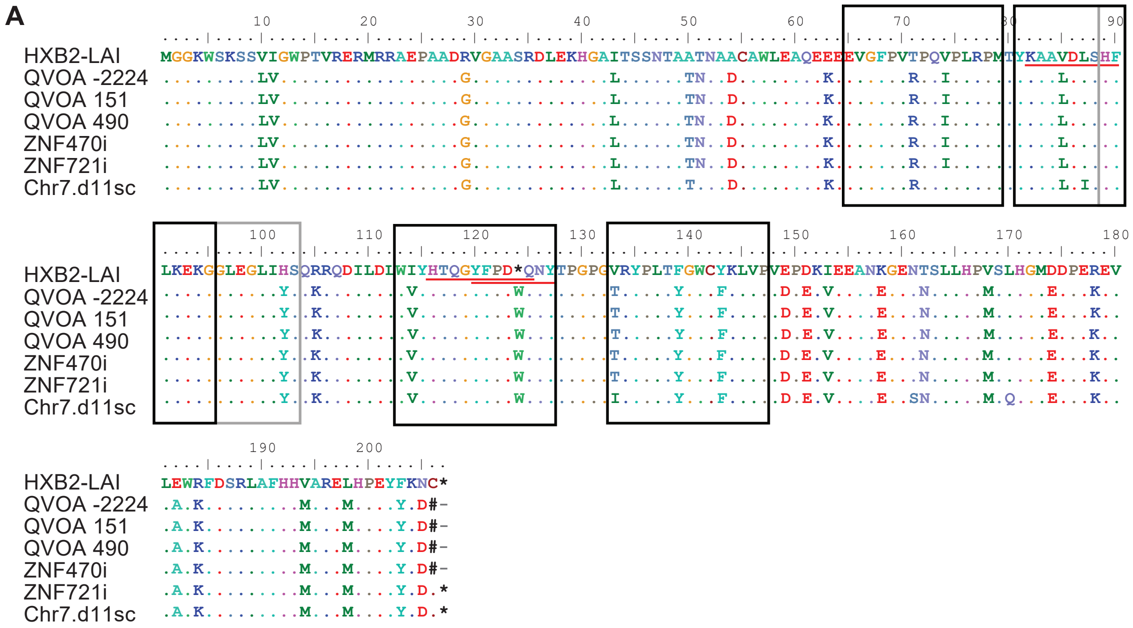

**Supplemental Figure S9. Nef protein sequence analysis of proviruses of interest.** (**A**) Nef protein sequences were codon aligned to the HXB2 reference; black boxes indicate targeted epitopes; grey box indicate overlapping epitope at positions 83-103; red lines indicate HLA-B*57–restricted epitopes; numbers next to QVOA sequences indicate days from the start of chemoradiation.

# Supplemental Table S1. Characteristics of previously published proviruses integrated into ZNF721/ABCA11P.

| Study | | Provirus information | | | | | | | | Participant characteristics | | | | | | | |
|---|---|---|---|---|---|---|---|---|---|---|---|---|---|---|---|---|---|
| Reference | Pubmed_id | Retrovirus Integration Database_id | Chromosome | Insert position* | Provirus orientation | *ZNF721* gene orientation | **Assays used** | **Evidence of intactness** | **Evidence of inducibility** | Participant id | Clinical notes | Age (years) | Sex | Race ethnicity | On ART | Years on ART | Plasma HIV RNA |
| This study | | | chr4 | 447418 | + | - | NFL proviral sequencing, LM-PCR/ISA, QVOA | Yes | induced by cognate antigen via PBMC stimulation | ES24 | EC on ART | 60 | M | African American | yes | 3 | suppressed |
| Guo 2022, Halvas 2020 | 36044447 | rid36044447_23888 | chr4 | 443311 | + | - | NFL proviral sequencing, LM-PCR/ISA, QVOA | Yes | inducible by QVOA, source of NSV | F07 | NSV | 59 | M | African American | yes | 19 | NSV |
| Guo 2022 | 36044447 | rid36044447_23886 | chr4 | 439930 | + | - | LM-PCR/ISA | unknown | | | | | | | | | |
| | | rid36044447_23887 | chr4 | 440816 | + | - | | | | | | | | | | | |
| | | rid36044447_23890 | chr4 | 446377 | + | - | | | | | | | | | | | |
| Guo 2022, Halvas 2020 | 36044447 | rid36044447_35276 | chr4 | 425766 | + | - | NFL proviral sequencing, LM-PCR/ISA, QVOA | Yes | inducible by QVOA | R09 | NSV | 73 | M | Caucasian | yes | 10 | NSV |
| Guo 2022 | 36044447 | rid36044447_35281 | chr4 | 434674 | - | - | LM-PCR/ISA | unknown | | | | | | | | | |
| | | rid36044447_35282 | chr4 | 441824 | + | - | | | | | | | | | | | |
| | | rid36044447_35284 | chr4 | 441923 | + | - | | | | | | | | | | | |
| | | rid36044447_35286 | chr4 | 462538 | + | - | | | | | | | | | | | |
| | | rid36044447_35294 | chr4 | 488671 | + | - | | | | | | | | | | | |
| | | rid36044447_10322 | chr4 | 425632 | + | - | | | | C03 | NSV | 43 | M | Caucasian | yes | 9 | NSV |
| | | rid36044447_10323 | chr4 | 439826 | + | - | | | | | | | | | | | |
| | | rid36044447_10324 | chr4 | 443536 | + | - | | | | | | | | | | | |
| | | rid36044447_10328 | chr4 | 449724 | + | - | | | | | | | | | | | |
| | | rid36044447_10329 | chr4 | 465001 | + | - | | | | | | | | | | | |
| | | rid36044447_10330 | chr4 | 466440 | + | - | | | | | | | | | | | |
| Eiunkauf 2019 | 30688658 | rid30688658_36 | chr4 | 442619 | + | - | MIP-seq | Yes | unknown | P1 | | 59 | M | | yes | 11.5 | suppressed |
| Jiang 2020, Lian 2021 | 32848246 | | chr4 | 444791 | + | - | MIP-seq | Yes | unknown | P9/EC9 | EC | | | | | | suppressed |
| Lian 2021, Sun 2023 | 34910552 | | chr4 | 448830 | + | - | MIP-seq, PHEP-seq | Yes | unknown | 27/P5 | | 62 | M | | yes | 14 | suppressed |
| Huang 2021 | 34636876 | rid34636876_9 | chr4 | 450716 | + | - | Q4PCR, MIP-seq | Yes | unknown | 5203 | | 59 | M | Caucasian | yes | 21 | suppressed |
| Coffin 2019 | 31217357 | rid31217357_2486 | chr4 | 443254 | + | - | LM-PCR/ISA | | | CH62-1 | Fiebig IV/V | | | | yes | 3.7 | suppressed |
| Coffin 2019 | 31217357 | rid31217357_3040 | chr4 | 457831 | + | - | LM-PCR/ISA | | | CH68-5 | Fiebig IV/V | | | | yes | 3.6 | suppressed |
| Bale 2021 | 33832973 | rid33832973_8584 | chr4 | 449365 | + | - | LM-PCR/ISA | unknown | | ZA011_preA | MTCT | 0.8 | F | | no | 0 | viremic |
| Bale 2021 | 33832973 | rid33832973_10090 | chr4 | 439658 | + | - | LM-PCR/ISA | | | ZA012_preA | MTCT | 0.4 | M | | no | 0 | viremic |
| Bale 2021 | 33832973 | rid33832973_10091 | chr4 | 440131 | + | - | LM-PCR/ISA | | | | | | | | | | |

## Supplemental Table S2. Oligonucleotides.

**Primers and probes used for specific and total virus quantification**

| Assay | Primer Name | PCR Reaction | Primer Type | Sequence (5'- 3') | Coordinates on HXB2 | Reference |
|---|---|---|---|---|---|---|
| ZNF721i Quantification | 721_IS_Fw | Digital PCR | Forward | TGTACTTTCCAAGATACCAGAA | 52-71 | This work |
| | 721_IS_Rev | Digital PCR | Reverse | GTAGCCTTGTGTGTGGTAGA | | |
| | 721_IS_Probe | Digital PCR | Probe | 5FAM/AGTATCTCT/ZEN/ATAGATACTGGAAGGGCT/3IABkFQ | | |
| ZNF470i Quantification | 470_IS_Fw | Digital PCR | Forward | 5HEX/CTTATTTAT/ZEN/TAATTGGAAGGGCTAATTTACTC/3IABkFQ | 52-71 | This work |
| | 470_IS_Rev | Digital PCR | Reverse | ATAACTTATCTGGGCTTTCCT | | |
| | 470_IS_Probe | Digital PCR | Probe | GTAGCCTTGTGTGTGGTAGA | | |
| Chr7.d11sc competition assay | U5PBS_delvar_Fw | Digital PCR | Forward | CTAGAGATCCCTCAGACCCTTT | 588-609 | This work |
| | U5PBS_delvar_Rev | Digital PCR | Reverse | ATTYGGCGTACTCACCAGTC | 739-758 | |
| | delvar_PSP | Digital PCR | Probe | 5HEX/CGAACAGGG/ZEN/ACTTGAAAACCAGAGGAGC/3IABkFQ | 643-681 | |
| | U5PBS_probe | Digital PCR | Probe | 5FAM/AAAGGGAAA/ZEN/CCAGAGGAGCTCTCTCGACAC/3IABkFQ | 663-692 | |
| Chr7.d11sc Quantification | Chr7.d11sc_IS_Fw | Digital PCR | Forward | ATCTGAGACAAGGCTCAATCAA | 58-89 | This work |
| | Chr7.d11sc_IS_Rev | Digital PCR | Reverse | GTTCTGCCAATCAGGGAAGTA | | |
| | Chr7.d11sc_IS_Probe | Digital PCR | Probe | 5FAM/CAGGAGGTC/ZEN/CTAATTGGAAGGGCTAATT/3IABkFQ | | |
| U5-R | RU5-F | Digital PCR | Forward | CTTAAGCCTCAATAAAGCTTGCC | 517-539; 9601-9623 | Anderson et al. (30253074) |
| | RU5-R | Digital PCR | Reverse | GGATCTCTAGTTACCAGAGTC | 577-597; 9661-9681 | |
| | RU5-probe | Digital PCR | Probe | 5FAM/AGTAGTGTG/ZEN/TGCCCGTCTG/3IABkFQ | 552-570; 9636-9654 | |

**Primers and probes used for specific TCR quantification**

| Assay | Primer Name | PCR Reaction | Primer Type | Sequence (5'- 3') | Reference |
|---|---|---|---|---|---|
| ZNF721i TCR Quantification | 721TCR_Fw | Digital PCR | Forward | GCACACAGCAGGAGGAC | This work |
| | 721TCR_Rev | Digital PCR | Reverse | GCTCACCCTCTCTCCCA | |
| | 721TCR_Probe1 | Digital PCR | Probe | 5FAM/CCCGAAGAA/ZEN/CTGCTCACCGC/3IABkFQ | |
| | 721TCR_Probe2 | Digital PCR | Probe | 5HEX/ATCTCTGTG/ZEN/CCAGCAGCCTTACAGG/3IABkFQ | |

**Primers used for cDNA synthesis, single genome sequencing (SGS) and sanger sequencing**

| Assay | Primer Name | PCR Reaction | Primer Type | Sequence (5'- 3') | Coordinates on HXB2 | Reference |
|---|---|---|---|---|---|---|
| cDNA synthesis/SGS | ES24_gag RO | outer | Reverse | TTAGCCTGTCTCTCAGTACAATC | 2062-2084 | This work |
| SGS/sanger sequencing | ES24_gag RN | nested/sanger | Reverse | TCATTTGGTGTCCTTCCTTTCC | 2038-2059 | |
| SGS | Delvar_4187_FO | outer | Forward | TGGGTACCAGCGCACAAAG | 4152-4170 | |
| SGS/sanger sequencing | Delvar_4210_FN | nested/sanger | Forward | GAATTGGAGGAAATGAACAAGTAG | 4171-4194 | |
| SGS | Delvar_5716_RO | outer | Reverse | TAGATATGTTGCCCTAAGCTATG | 5676-5698 | |
| SGS/sanger sequencing | Delvar_5687_RN | nested/sanger | Reverse | TAGCCTAGGAAAATGTCTAACAGC | 5646-5669 | |
| SGS | U5_gag_FO | outer | Forward | GTARCTAGAGATCCCTCAGAC | 583-603 | Simonetti et al. (33301425) |
| SGS/sanger sequencing | U5_gag_FN | nested/sanger | Forward | AAATCTCTAGCAGTGGCGCC | 621-640 | |
| SGS | env_FO | outer | Forward | GCCAGTAGTRTCAACYGAA | 6979-6997 | |
| SGS/sanger sequencing | env_FN | nested/sanger | Forward | CTGCTAAATGGCAGTCTAGC | 7001-7020 | |
| SGS | env_RO | outer | Reverse | GCARATGAGTTTTCYAGAGCA | 8015-8035 | |
| SGS/sanger sequencing | env_RN | nested/sanger | Reverse | TTGCCTGGAGCTGYTTRATGC | 7938-7958 | |
| Sanger sequencing | INT3A2 | sanger | Reverse | AGCTTCCTCATTGATGGTCTCTTT | 1393-1416 | Einkauf et al. (30688658) |
| SGS | 5CP1 | outer | Forward | GAAGGGCACACAGCCAGAAATTGCAGGG | 1981-2008 | |
| SGS/sanger sequencing | 2.5 | nested/sanger | Forward | CCTAGGAAAAAGGGCTGTTGGAAATGTGG | 2011-2039 | |
| SGS | RT3.1 | outer | Reverse | GCTCCTACTATGGGTTCTTTCTCTAACTGG | 3830-3859 | |
| SGS/sanger sequencing | RT3798R | nested/sanger | Reverse | CAAACTCCCACTCAGGAATCCA | 3777-3798 | |
| SGS | RT3597mixF | outer | Forward | AAAACAGGAAARTATGCAA | 3597-3615 | |
| SGS/sanger sequencing | RT3626F | nested/sanger | Forward | TGCCCACACTAATGATGTAA | 3626-3645 | |
| SGS | SC05R | outer | Reverse | AGCTCTTCGTCGCTGTCTCCGCTT | 5980-6003 | |
| SGS/sanger sequencing | SC02R | nested/sanger | Reverse | CTTCCTGCCATAGGAGATGCCTA | 5957-5979 | |
| SGS | VP5450F | outer | Forward | CAGGACATAACAAGGTAGGATC | 5450-5471 | |
| SGS/sanger sequencing | VP5549F | nested/sanger | Forward | AGAGGATAGATGGAACAAGCCCCAG | 5550-5574 | |
| SGS | CO602 | outer | Reverse | GCCCATAGTGCTTCCTGCTGCTCCCAAGAACC | 7785-7816 | |
| SGS/sanger sequencing | V3CR | nested/sanger | Reverse | TGCTCTTTTTTCTCTCTSCACCACT | 7735-7759 | |

**Primers used to confirm integration site**

| Assay | Primer Name | PCR Reaction | Primer Type | Sequence (5'- 3') | Coordinates on HXB2 | Reference |
|---|---|---|---|---|---|---|
| ZNF470i | ZNF470_FO | outer | Forward | CAGTAGCGCTACTGTTTCTAATTC | | This work |
| | ZNF470_FN | nested/sanger | Forward | CTACATATAACTTATCTGGGCTTTCC | | |
| | ZNF470_RO | outer | Reverse | GTTACCCAGGCTGGAGTTGAA | | |
| | ZNF470_RN | nested/sanger | Reverse | CACACCTGGCCCTACTTTCAGT | | |
| ZNF721i | ZNF721_FO | outer | Forward | AATTTTATGTACTTTCCAAGATACCAG | | This work |
| | ZNF721_FN | nested/sanger | Forward | TACCAGAAAAAAGTATCTCTATAGA | | |
| | ZNF721_RO | outer | Reverse | TTTGTACTGATACACCTTTACTTCT | | |
| | ZNF721_RN | nested/sanger | Reverse | GGCGTATCTGCTAGAGATTTTC | | |
| Chr7.d11sc | Delvar_FO | outer | Forward | CCATGCCTGTTAGAGAGTGTG | | This work |
| | Delvar_FN | nested/sanger | Forward | GAGACAAGGCTCAATCAATTTAG | | |
| primers annealing to HIV | ES24_gag RO | outer | Reverse | TTAGCCTGTCTCTCAGTACAATC | 2062-2084 | Simonetti et al. (33301425) |
| | ES24_gag RN | nested/sanger | Reverse | TCATTTGGTGTCCTTCCTTTCC | 2038-2059 | |
| | env_FO | outer | Forward | GCCAGTAGTRTCAACYGAA | 6979-6997 | |
| | env_FN | nested/sanger | Forward | CTGCTAAATGGCAGTCTAGC | 7001-7020 | |
